# Psycho-educational interventions for children and young people with Type 1 Diabetes in the UK: How effective are they? A systematic review and meta-analysis

**DOI:** 10.1371/journal.pone.0179685

**Published:** 2017-06-30

**Authors:** Dimitrios Charalampopoulos, Kathryn R. Hesketh, Rakesh Amin, Veena Mazarello Paes, Russell M. Viner, Terence Stephenson

**Affiliations:** Great Ormond Street Institute of Child Health, University College London, London, United Kingdom; University of Nottingham School of MedicineD, UNITED KINGDOM

## Abstract

**Aims:**

To synthesise evidence from UK-based randomised trials of psycho-educational interventions in children and young people (CYP) with Type 1 Diabetes (T1D) to inform the evidence-base for adoption of such interventions into the NHS.

**Methods:**

We searched Medline, Embase, Cochrane, PsycINFO, CINAHL, and Web of Science up to March 2016. Two reviewers independently selected UK-based randomised trials comparing psycho-educational interventions for improving management of T1D for CYP with a control group of usual care or attention control. The main outcome was glycaemic control measured by percentage of glycated haemoglobin (HbA_1c_); secondary outcomes included psychosocial functioning, diabetes knowledge, adverse and other clinical outcomes. A narrative synthesis and meta-analysis were conducted. Pooled effect sizes of standardised mean difference (SMD) were calculated.

**Results:**

Ten eligible trials of three educational and seven psycho-educational interventions were identified. Most interventions were delivered by non-psychologists and targeted adolescents with more than one year duration of diabetes. Meta-analysis of nine of these trials (N = 1,838 participants) showed a non-significant reduction in HbA_1c_ attributable to the intervention (pooled SMD = -0.06, 95% CI: -0.21 to 0.09). Psycho-educational interventions aiming to increase children’s self-efficacy had a moderate, beneficial effect (SMD = 0.50, 95% CI: 0.13 to 0.87). No benefits on diabetes knowledge and other indicators of psychosocial functioning were identified.

**Conclusions:**

There is insufficient evidence to recommend the use of particular psycho-educational programme for CYP with T1D in the UK. Further trials with sufficient power and reporting standards are needed. Future trials could consider active involvement of psychological specialists in the delivery of psychologically informed interventions and implementation of psycho-educational interventions earlier in the course of the disease.

**Systematic review registration:**

PROSPERO CRD42015010701

## Introduction

Type 1 Diabetes (T1D) is one of the most common chronic diseases in childhood and adolescence, with an incidence of 28.2 new cases per 100,000 children under the age of 14 in the United Kingdom (UK) every year [1]. The UK has the fourth largest paediatric diabetes population in Europe and the fifth largest paediatric diabetes population in the world [2, 3] with the most recent estimates indicating at least 29,000 children under 19 years have T1D in the country [4, 5].

In the UK, children and young people (CYP) with T1D are usually managed by multi-disciplinary teams in hospital-based diabetes clinics. T1D management primarily aims to optimise glucose control, whilst also maintaining quality of life. The gold standard for assessing average glucose control over the preceding 2–3 months is glycated Haemoglobin A_1c_ (HbA_1c_) and regular testing is recommended to guide management advice. The National Institute for Health and Care Excellence (NICE) has recently recommended a target for HbA_1c_ of 6.5% (48 mmol/mol) or lower [6]. Although it is widely accepted that intensive management aiming for lower glycaemic targets confers a significant reduction in risk of diabetes-related complications [7], only 6.4% of children cared for in clinical services in England and Wales meet this target [5].

Although the mainstay of T1D management is through insulin and dietary modifications, the need for structured educational programs at diagnosis has been highlighted as a priority by government bodies and diabetes organisations in the UK [6, 8, 9]. Such programs constitute an integral part of diabetes management since they are necessary to integrate the complex demands of diabetes self-management into daily life. However, it is well accepted that education is a necessary, but not sufficient component of diabetes care. A distinction has been made between traditional education programs that aim to teach diabetes-related knowledge and skills, and those that incorporate psychological elements and provide support in areas such as problem-solving, goal-setting, stress management, coping, motivation, and counselling. Although a successful educational programs have been introduced across the UK for adults with T1D [10, 11], there is a lack of evidence-base for equivalent programs for children and adolescents with no agreed standardised package available in the UK [12].

Over the last few years several systematic reviews have examined the effect of these programs on metabolic and psychological outcomes in CYP with T1D. In a review commissioned by the NHS Health Technology Assessment programme in 2001, Hampson et al. made the first comprehensive attempt to systematically review literature on the effectiveness of psycho-educational interventions among adolescents [13]. They summarised intervention effects using the standardised mean difference (SMD) (i.e. difference in mean change-from-baseline scores between groups divided by the pooled standard deviation) which allows for a direct comparison across trials that used different scales to assess outcomes. They concluded that psycho-educational interventions had a small, non-significant effect on glycaemic control corresponding to a decrease of 0.6% in HbA_1c_ (SMD = 0.3, 95% CI -0.04 to 0.7) but appeared to confer more substantial improvements in psychological outcomes (SMD = 0.4, 95% CI 0.2 to 0.6) [13]. The review also highlighted that evidence was predominantly derived from the USA with a notable shortage of UK-based randomised controlled trials (RCTs). An updated review by Murphy et al. in 2006 showed little progress in the development of new interventions in the UK [14]. Two subsequent meta-analyses provided evidence for a glycaemic benefit of such interventions. The first showed that children and adolescents who received a psychological intervention had reduced HbA_1c_ levels (SMD = -0.35, 95% CI -0.66 to -0.04) and psychological distress (SMD = -0.5, 95% CI: -0.8 to -0.1) compared to controls [15]. The second meta-analysis focused on family-based psycho-educational interventions and found a beneficial effect on both glycaemic control (mean difference in % HbA_1c_ = -0.6, 95% CI -1.2 to -0.1) and diabetes knowledge (SMD = 0.94, 95% CI 0.67 to 1.82) [16].

Evidence for the effectiveness of psycho-educational interventions in children with T1D is predominantly derived from non-UK trials. Only two, small scale RCTs conducted in the UK were included in previous reviews [17, 18], the most recent of which was published in 2002 [17]. Yet, the evidence for the effectiveness of such interventions might be context-dependent since, for example, the quality of standard care against which interventions are compared shows considerable variation between countries [19]. This suggests that the extent to which conclusions from previous reviews can be generalised to the UK health care system is unclear. Moreover, the last decade has also seen a number of large UK-based RCTs of psycho-educational interventions which have not been systematically reviewed. A need, therefore, exists for a comprehensive assessment of these interventions to determine whether there is sufficient evidence to support adoption of psycho-educational interventions into the NHS.

This systematic review aims to critically appraise and synthesise evidence from UK-based RCTs on the effectiveness of psychoeducational interventions in improving glycaemic control, psychosocial functioning, diabetes knowledge and other outcomes in CYP with T1D. It is expected that findings of this review will be used to inform the evidence-base for adoption of such interventions into the NHS.

## Methods

The protocol for this review has been published in the International Prospective Register for Systematic Reviews (PROSPERO) (Registration number: CRD42015010701 –see S1 File). The conduct and report of the current systematic review is in accordance with the Preferred Reporting Items of Systematic Reviews and Meta-Analysis (PRISMA) guidelines (see S1 Checklist) [20].

### Search strategy

Six databases (Medline, Embase, Cochrane, PsycINFO, CINAHL, and Web of Science) were systematically searched for relevant citations published up until March 2016. The search strategy was developed with the assistance of a professional librarian. A combination of free-text words and medical subject heading (MeSH) terms were used to generate five subsets of citations relating to population, intervention, outcomes of interest, randomised controlled trials and studies conducted in the UK (see S2 File). Results were limited to CYP up to 24 years. The search was not limited by language or year of publication. A number of “snowballing” techniques were also used to minimise the potential of publication bias and to increase the sensitivity of our search. These included hand-searching reference lists of all selected articles, and contacting experts and corresponding authors of selected articles for any known published or unpublished relevant trials.

### Eligibility criteria

We included trials conducted in the UK that examined the effectiveness of educational or psycho-educational intervention in CYP up to 24 years with T1D. A broad definition of psycho-educational interventions was used; we included interventions targeting CYP, their families and/or health care professionals that aimed to improve management of diabetes in children. Interventions including any type of teaching diabetes-related knowledge or skills and/or providing any form of psychosocial training or support were eligible. Studies were not excluded based on setting, delivery or duration of the intervention. Interventions had to be randomised controlled trials that involved a non-intervention arm of children with T1D receiving standard care. Trials in which the control group was matched for the extra contact time (attention control) were also included. Studies combining type 1 and type 2 diabetes or children and young people (≤24 years old) with adults (>24 years) were excluded unless results were stratified by type of diabetes or age group respectively. Finally, we excluded letters, commentaries, editorials, reviews, conference proceedings, intervention development protocols, pilot trials and qualitative studies.

### Types of outcome measures

The primary outcome of interest was glycaemic control, as measured by levels of HbA_1c_. Secondary outcomes included indicators of psychosocial functioning, diabetes knowledge, insulin regimen, adverse events (episodes of hypoglycaemia and diabetes ketoacidosis-DKA), and service utilisation.

### Study selection and data extraction

Retrieved citations were entered into a reference management library (EndNote), and duplicates were removed. Initially, titles and abstracts of unique citations were screened and full texts of potentially eligible articles were then retrieved and screened. Titles, abstracts, and full texts were independently reviewed by 2 reviewers (DC and KRH). In parallel, the same reviewers then independently extracted data from all eligible trials using a pre-piloted data extraction form (see S3 File) as per guidelines by the Centre for Review and Dissemination (CRD) for systematic reviews in healthcare [21]. At all stages, any discrepancies were resolved by joint discussion. We extracted data on study design and methodology, intervention characteristics and type of care received by controls. We also extracted data on sample size, baseline characteristics, recruitment and study completion rates, reasons for attrition, power of the study, baseline and follow-up outcome data for each trial arm, and information for assessment of the risk of bias. Corresponding authors of included studies were contacted by email for clarification on trial methods or data whenever there was insufficient data reported (three authors were contacted, all provided further information).

Interventions were categorised according to their primary methodology as educational (i.e. those targeting diabetes-related knowledge and skills), psychological (i.e. those providing any form of psychosocial support) or psycho-educational (those combining educational with psychological elements). Psycho-educational interventions were further classified into the following categories: supportive or counselling therapy (including motivational interviewing, non-directive counselling, and solution-focused therapy); cognitive behavioural therapy (including techniques such as goal setting, activity scheduling, problem solving, cognitive restructuring, and stress management); family systems therapy; psychotherapy (including psychodynamic or interpersonal approaches) and other interventions (including eclectic approaches).

### Quality assessment

Quality assessment was conducted independently by two reviewers (DH and KRH) and disagreements were resolved by consensus. Quality of individual trials was assessed using six domains of the Cochrane Collaboration’s tool for assessing risk of bias [22], including sequence generation; allocation concealment; blinding of outcome assessors; completeness of outcome data; selective reporting of outcomes; and other sources of bias. Since blinding of participants and personnel to knowledge of the intervention was not possible, this domain was excluded from the assessment. Assessment of the two domains relating to blinding of outcome assessors and data completeness was made separately for glycaemic and psycho-educational outcomes. For each domain, studies were classified as being at low, high or unclear risk of bias.

### Data synthesis and calculation of effect sizes

Data were analysed through narrative synthesis and meta-analysis. We used the SMD to summarise intervention effects on continuous outcomes, calculated by dividing the between group difference in mean change-from-baseline scores (or follow-up scores adjusted for baseline values) by the pooled standard deviation of the change scores [23]. We calculated the intervention effect using the follow-up interval set *a priori* for the definition of the primary outcome. Four trials provided multiple follow-up measurements without stating any primary time point, in which case we used the longest follow-up measurement available. To examine whether results were sensitive to selection of time point, we repeated the meta-analyses, where possible, by using the shortest follow-up measurement that was available immediately after the end of the intervention; no differences in the summary estimates were observed (see S4 File). If standard deviations of change scores were not available from the published report, we obtained them by correspondence with the authors, or by hand calculating on the basis of available published data. For seven trials none of the above was feasible and standard deviations of change scores were imputed assuming a conservative correlation coefficient of 0.5 [24]. We varied the assumed correlation of r = 0.5 between baseline and follow-up measurements from r = 0.3 to r = 0.7 to see if this has any effect on the summary estimates; results were robust to these variations.

For trials with multiple intervention arms, the intervention arm which was directly comparable to the control arm (i.e. without any co-intervention or change in routine care) was chosen. In cross-over trial designs we only used data from the first period. For cluster-randomised trials we used effect sizes adjusted for clustering effect and baseline values, or if not available, we adjusted sample sizes for the “design effect” [23].

To avoid unit of analysis errors, each trial contributed only one estimate per psychosocial construct. For example, where studies reported both patient and parent/carer reports of the same measure the former were used in the meta-analysis. Moreover, if studies reported multiple comparisons for different participants (such as for younger and older children), these measures were combined within each study before being entered in the meta-analysis. Finally, for comparisons that were not independent of one another (such as when studies reported several dimensions of quality of life for the same participants), we calculated a synthetic effect size for each study. This was defined as the weighted mean of the multiple effects with a variance that takes account of the correlation between the outcomes [25], again assuming it to be r = 0.5 if not stated.

### Calculating overall summary effects

We combined effect sizes from individual studies using a random effects model to account for differences in the interventions and settings across studies. Results were provided as pooled SMD with 95% confidence intervals. A standardised mean difference of ~0.2, ~0.5, and ~0.8 was considered as small, medium and large respectively [26]. To facilitate clinical interpretation of intervention effects on glycated haemoglobin, we re-expressed the pooled SMD into absolute units by multiplying the estimate by the pooled standard deviation of all included studies. We generated forest plots, sorted by level of precision, to visually assess intervention effects across studies. All analyses were performed using STATA 12 (StataCorp, College Station, Texas).

### Assessment of heterogeneity and publication bias

Heterogeneity between studies was assessed by the I^2^ statistic, which quantifies the percentage of total variation that can be attributed to heterogeneity [27, 28]. Values of I^2^ ≤50%, 50–75%, and ≥75% were considered as indicative of low, moderate and high heterogeneity respectively [27]. Individual studies were removed one at a time from the meta-analysis to explore whether heterogeneity could be reduced. We also investigated potential sources of heterogeneity by conducting subgroup analyses, where possible, against potentially modifying factors (type of intervention, study quality and age group). Funnel plot was constructed to explore the possibility of publication bias for the primary outcome.

## Results

**Fig 1:**
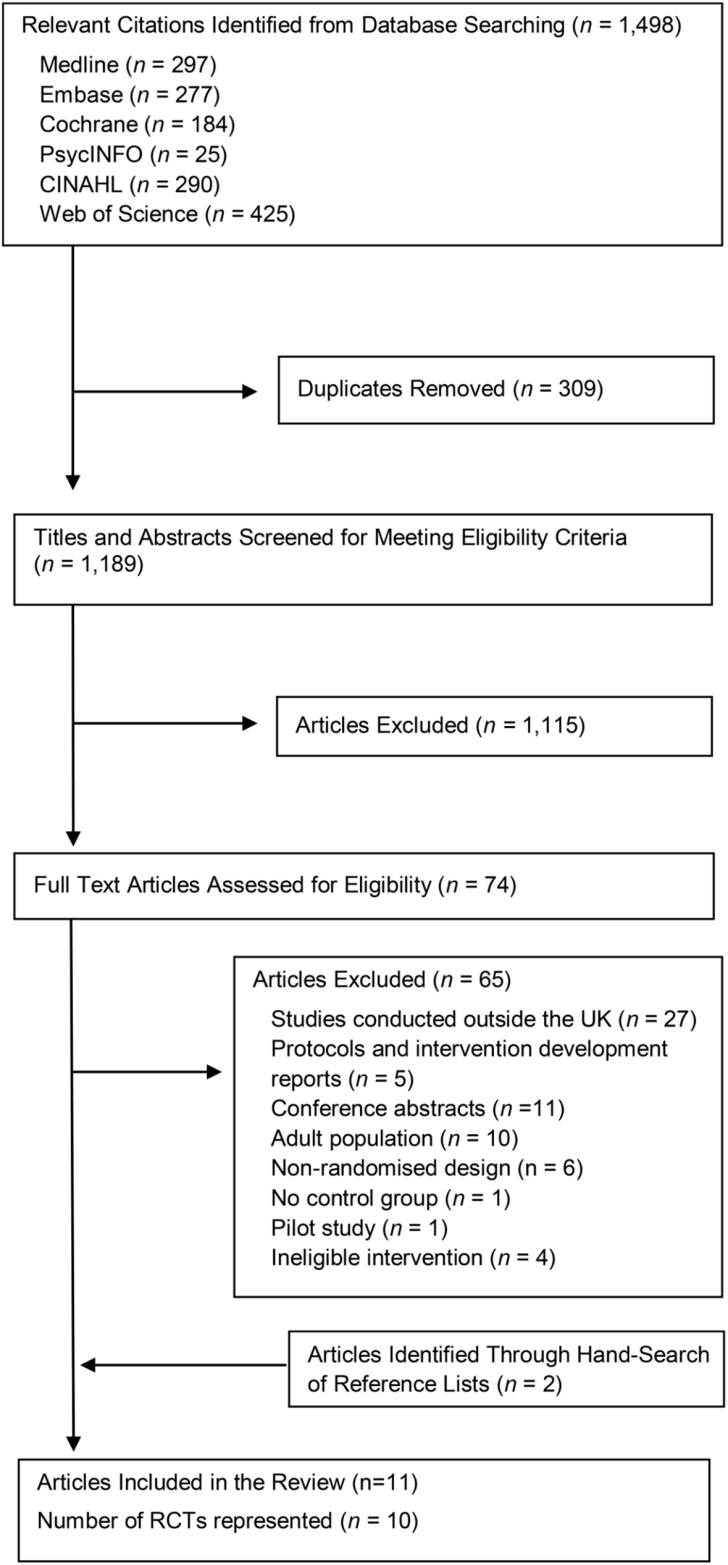
Flow diagram of study selection.

The search strategy yielded 1,189 potentially relevant papers, of which 74 were read in full. Two additional articles were identified from reference lists. As per the eligibility criteria, we excluded a small pilot trial which examined the feasibility of a UK psychoeducational intervention [29]. Results of the same intervention were reported in a subsequent main trial which was included in the current review [30]. In total, eleven studies [17, 18, 30–37] representing ten randomised controlled trials were found to meet the eligibility criteria and were included in the current review (see Fig 1).

Characteristics of included RCTs are shown in Table 1 and S5 File. In all RCTs participants were analysed by intention to treat. Six trials had a parallel group design [17, 30, 31, 33–35], one trial had a cross-over design [18] and three were cluster randomised [32, 36, 37]. Sample sizes ranged from 48 to 693 with a median of 113. Participation rates were generally low, ranging from 31% to 70.2% (median 50%). Six studies recruited only adolescents [17, 30, 31, 33, 34, 36] one of which also included young people up to 24 years [17]. All but three trials [17, 18, 34] targeted children who had been diagnosed with T1D for more than one year. Median duration of diabetes was 5.6 years and ranged from 2.8 to 9.2 years.

**Table 1:** Characteristics of included trials.

First author (publication year)	Country (study name)	No of participants randomised (eligibility criteria)	Mean (SD) % HbA_1c_ at baseline	Mean (SD or range) duration of diabetes (years)	Mean (SD or range) age (years)	Intervention, setting, mode of delivery	Theoretical Model	Control group	Interventionist	Duration of intervention in months (except as noted)	Assessment points ^a^ (months)	Time in min spent on each session (No of sessions)
Bloomfield (1990)	Scotland	48 (children <13 years with T1D > 3 months	9.3 (1.5)	2.8 (2.1)	9.0 (3.0)	Semi-structured educational program, Community, Group of families	-	Usual care	D	12	12	210 (10)
Howells (2002)	Scotland	79 (children 12–24 years)	8.8 (1.7)	7.0 (4.5)	16.8 (3.4)	Negotiated telephone support, Home, Child	SLT	Usual care	D	12	12	9 (16)
Franklin (2006)	Scotland (Sweet Talk)	64 (children 8–18 years with T1D >1 year)	10.2 (1.7)	4.1 (1.7–8.6)	13.5 (10.5–15.6)	Automated text message support plus goal-setting education, Home, Child	SCT	Usual care	MDT	12	12	NA
Channon (2007)	Wales	80 (adolescents 14–17 years with T1D >1 year)	9.2 (1.9)	9.2 (1.8)	15.3 (1.1)	Motivational interviewing, Home & community, Child	MSA	Usual care plus additional support visits	PSY + N	12	6, 12, 24	20–60 (4)
Murphy (2012)	UK (FACTS)	305 (adolescents with T1D >1 year)	9.3 (1.9)	5.6 (3.4)	13.2 (2.0)	Family-cantered structured program, Clinic, Group of families	SLT	Usual care	MDT	6	9, 12, 18	90 (6)
Robling (2012)	UK (DEPICTED)	693 (children 4–15 years with T1D >1 year)	9.3 (1.8)	5.1 (2.7)	10.6 (2.8)	Training healthcare practitioners in consultation skills using eclectic approach, Clinic, Child with carer	CMCS	Usual care	MDT	12	12	100 (3.5)
Coates (2013)	N. Ireland (CHOICE)	135 (adolescents 13–19 years with T1D >1 year)	8.9 (1.5)	6.6 (3.8)	15.4 (1.8)	Structured educational program, Clinic, Group of families	-	Usual care	N + D	5	1, 3, 6, 12, 24	180 (4)
Doherty (2013)	UK (Triple P)	90 (Parents of adolescents aged 11–17 years)	8.5 (1.3)	5.1 (3.4)	13.5 (1.0)	Self-directed, web-based behavioural intervention, Home, Parents	SLT	Usual care	NA	2.3	2.3	60 (10)
Christie (2014)	England (CASCADE)	365 (Children 8–16 years with T1D >1 year & HbA_1c_ ≥ 8.5%)	10.0 (1.5)	5.9 (3.3)	13.2 (2.1)	Motivational interviewing, solution-focused brief therapy, Clinic, Group of families	MSA	Usual care	N + O	4	12,24	120 (4)
Price (2016)	UK (KICk-OFF)	480 (adolescents 11–16 years with T1D > 1 year)	9.2 (1.7)	5.6 (2.0)	13.8 (1.5)	Intensive, structured education course, Community, Group of children	-	Usual care	N + D + O	5 days	6, 12, 24	420 (5)

Notes: NA: not applicable, D: dietitian, PSY: psychologist, N: nurse, MDT: multidisciplinary team member, O: other, FACTS: Families, Adolescents, and Children Teamwork Study, DEPICTED: Development and Evaluation of a Psychosocial Intervention in Children and Teenagers Experiencing Diabetes, CHOICE: Carbohydrate, Insulin, Collaborative Education, CASCADE: Child and Adolescent Structured Competencies Approach to Diabetes Education, KICk-OFF: Kids In Control OF Food, SLT: Social Learning Theory, SCT: Social Cognitive Theory, MSA: Menu of Strategies Approach, CMCS: Consultation Model of Communication Styles

^a^ from start of intervention

Of the ten RCTs, seven [17, 30–37] used psycho-educational and three [18, 33, 36] purely educational interventions. Six trials [30, 32–35, 38] provided reference to the full trial protocol. However, in only four trials [30, 32, 35] was the intervention described in sufficient detail to be replicated in practice. In all RCTs, control groups received standard care which in most cases included three to five clinic visits per year; however, in one trial [31] the control group was matched for contact time by receiving additional support visits. Only one trial [32] provided a detailed description of standard care.

All psycho-educational interventions reported using an underlying theoretical model. Of the seven psycho-educational interventions, three used supportive or counselling therapy [31, 32, 35], two employed cognitive behaviour therapy strategies [17, 34], one used family therapy [30], and one [37] used an eclectic approach. Interventions targeted individual children [17, 31, 35, 37], groups of children [36], family groups [18, 30, 32, 33], and parents [34]. Four interventions [30, 32, 33, 38] were delivered in clinics and six [17, 18, 31, 34–36] in home or other community settings. Intensity of interventions varied considerably with total time spent on intervention ranging from 2.4 to 35 hours (median of 8.5 hours). Most interventions were delivered by dietitians and nurses and in only one trial [31] the interventionist had a background in psychology. Evidence for training of the interventionist was provided in half of the trials [17, 30, 32, 36, 37].

Five interventions [17, 18, 31, 35, 37] had a duration of one year with the remaining interventions [30, 32–34, 36] lasting for 6 months or less. Half of the trials had a follow-up assessment after the end of the intervention. Retention rates ranged from 43% to 100% and half of the trials [18, 31, 33–35] were deemed underpowered to detect an effect in their primary outcome. Six trials reported monitoring adherence to trial protocol [17, 31, 32, 34, 36, 37]. Eight trials [17, 18, 30, 32–34, 36, 37] provided information on intervention attendance and in three of them [30, 32, 34] attendance rates were considered as potentially insufficient to demonstrate an intervention effect (see S5 File).

### Risk of bias

**Fig 2:**
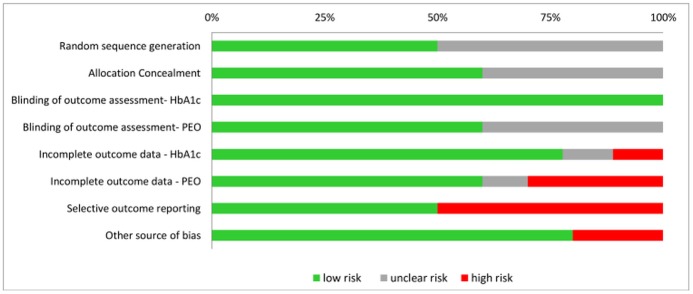
Outcome of risk of bias assessment by type of bias (Note: PEO = psycho-educational outcomes).

Risk of bias assessment is presented in Fig 2. Risk of selection bias due to inadequate sequence generation was unclear in half of the trials [18, 31–33, 37] since method of randomisation was not reported by authors. Risk of bias due to poor allocation concealment could not be assessed in four trials [18, 30, 32, 35]. Although blinding of participants and interventionists is not feasible in the context of psycho-educational interventions, risk of detection bias from outcome assessment was considered small for HbA_1c_ (objectively measured) and for most of the psycho-educational outcomes (use of standardised scales). There was high risk of bias due to incomplete psychological data in three trials [30, 31, 34], which reflected the high attrition rate in this type of interventions. Five trials [18, 31, 33, 36, 37] did not report all psychological outcomes and were at high risk of selective outcome reporting. Other sources of bias included baseline imbalances not accounted for in the analyses [34] and inappropriate study design (cross-over) [18]. When all bias domains were considered together, one trial [18] scored low risk in only one domain, three trials [30, 31, 33] scored low risk in two or three bias categories, and the remaining studies [17, 32, 34–37] scored low risk in four or more domains (see S6 File).

### Effectiveness of interventions

#### Glycated haemoglobin (HbA_1c_)

**Fig 3:**
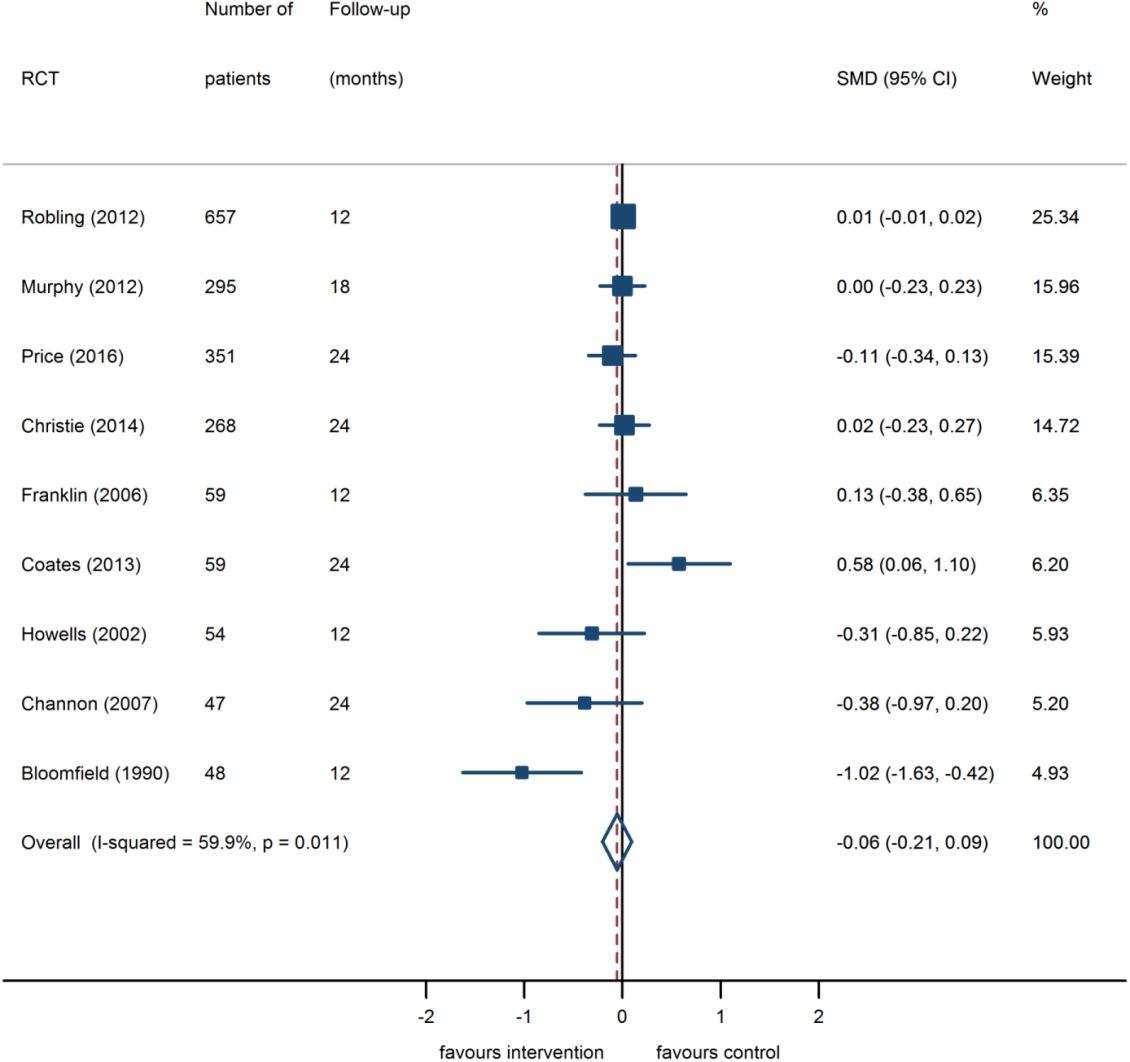
Random effects meta-analysis of change scores in HbA1c (%) in psycho-educational intervention group compared with control group. Intervention effects calculated as Standardised Mean Difference (SMD) with 95% confidence interval. A negative effect indicates improved glycaemic control attributable to intervention.

A total of nine RCTs [17, 18, 30–33, 35–37] including 1,838 participants assessed the effectiveness of educational and psycho-educational interventions in reducing HbA_1c_ levels and were included in the meta-analysis. Effect sizes in four out of the nine trials showed a reduction in HbA_1c_ levels attributable to the intervention (see Fig 3). The pooled analysis did not show a statistically significant glycaemic benefit (pooled SMD = -0.06, 95% CI: -0.21 to 0.09). The intervention effect was equivalent to a reduction in HbA_1c_ of 0.1% (95% CI: -0.4% to 0.2%). There was moderate heterogeneity between the studies (I^2^ = 59.9%), which was fully explained by an early trial of an educational intervention [18] with a low methodological quality rating. Exclusion of this trial from the meta-analysis did not change the overall conclusion (SMD = -0.02, 95%CI: -0.13 to 0.09, I^2^ = 0%). The intervention effect on HbA_1c_ remained non-significant when subgroup analyses were performed for purely educational interventions (SMD = -0.17, 95% CI -0.88 to 0.55, three studies pooled), psycho-educational interventions (SMD = 0.01, 95% CI: -0.01 to 0.02, six studies pooled), or interventions targeting adolescents (SMD = -0.05, 95% CI -0.20 to 0.10, four studies pooled).

#### Psychosocial functioning

**Fig 4:**
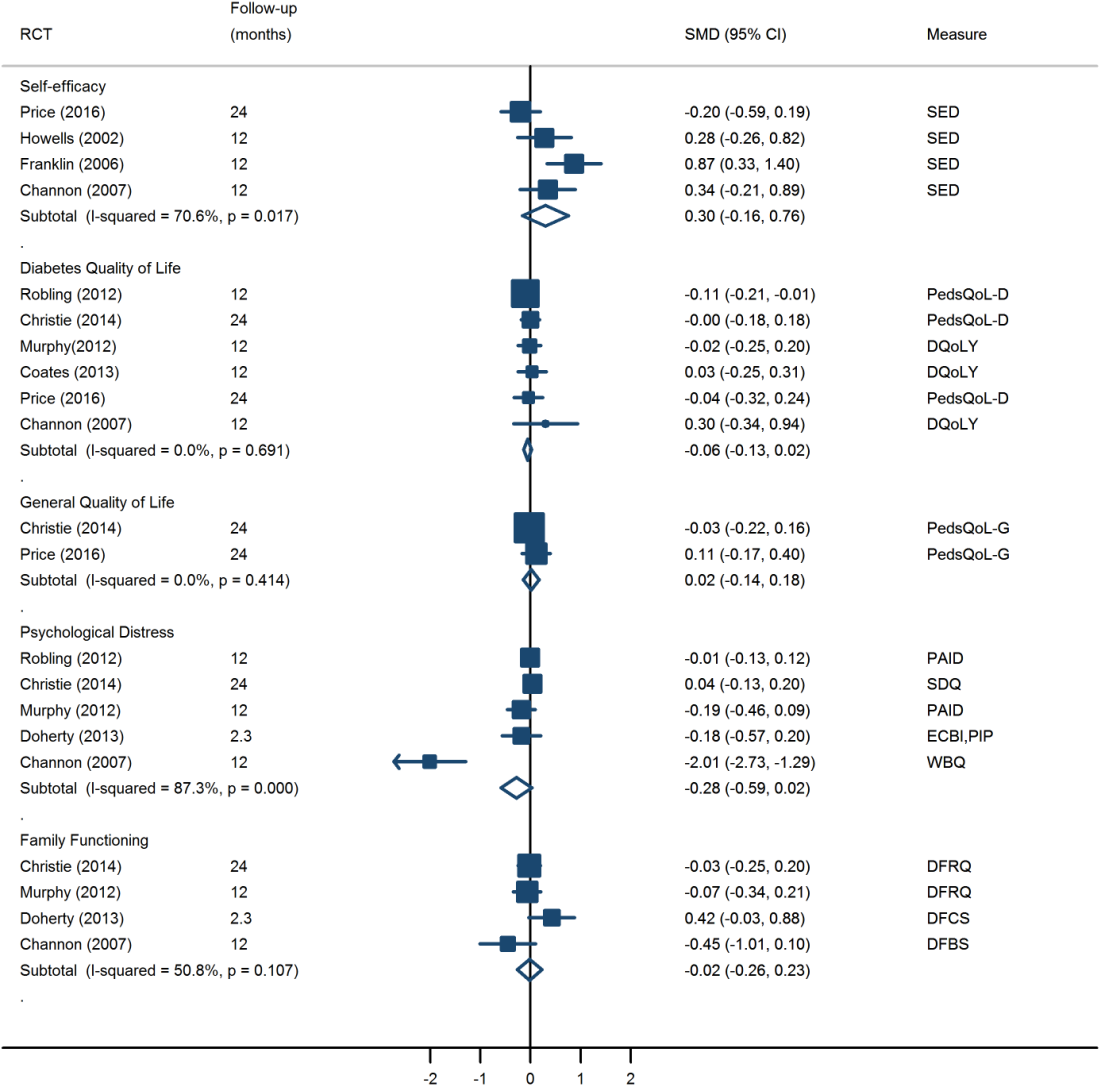
Intervention effects on psychosocial outcomes calculated as Standardised Mean Difference (SMD) of change scores with 95% confidence interval. **A positive effect in quality of life, self-efficacy, and family functioning and a negative effect is psychological distress favour intervention. The diamonds show the pooled SMD based on random effects model.** Notes: SED = Self-efficacy for diabetes; PedsQoL-D = Paediatric quality of life inventory: diabetes module; DQoLY = Diabetes Quality of life measure for youths (reverse scaling); PedsQoL-G = Paediatric quality of life inventory: generic scale; PAID = Problem Areas in Diabetes scale; SDQ = Strengths and difficulties Questionnaire- impact score; ECBI = Eyberg child behavior inventory; PIP = Paediatric Inventory for parents; WBQ = Well-being questionnaire (reverse scaling); DFRQ = Diabetes Family Responsibility Questionnaire (dyadic score in Christie (2014) and parental report in Murphy (2012)); DFCS = Diabetes family conflict scale; DFBS = Diabetes Family Behavior scale.

Interventions addressed various measures of psychosocial functioning (see S7 File). Four trials of one educational [36] and three psycho-educational interventions [17, 31, 35] measured the effect of interventions on increasing self-efficacy. Overall, interventions produced a small, non-significant improvement in self-efficacy (SMD = 0.30, 95% CI: -0.16 to 0.76, I^2^ = 70.6%). Heterogeneity was reduced when we removed the one educational intervention [36]; when it was omitted, effect of psycho-educational interventions on self-efficacy increased in magnitude and became statistically significant (SMD = 0.50, 95% CI: 0.13 to 0.87, I^2^ = 27.8%). There was no evidence for a beneficial effect of psycho-educational interventions on other indicators of psychosocial functioning, including diabetes-specific quality of life, general quality of life, psychological distress and family functioning (see Fig 4). Some other psychosocial outcomes were explored in isolation and showed no significant changes between the groups; these included locus of control [31], patient empowerment [33], health care climate [37], and patient enablement [37].

#### Diabetes knowledge

Five trials (one educational [18] and four psycho-educational [17, 31, 32, 35]) measured diabetes-related knowledge using similar scales [39–41]. Four trials [17, 18, 31, 35] provided sufficient data for the meta-analysis. With a random effects model, psycho-educational interventions were associated with a non-significant reduction in diabetes knowledge, in all cases measured immediately after the end of the intervention (SMD = -0.11, 95% CI: -0.45 to 0.23, I^2^ = 40.5%). Heterogeneity between studies was fully explained by an early trial of an educational intervention which was the only one to show a beneficial effect [18]. One study could not be pooled in the meta-analysis but reported no difference in post-intervention knowledge scores between the two groups [32].

#### Adverse and other outcomes

Seven trials [17, 30, 32, 33, 35, 36, 38] reported on the incidence of DKA and hypoglycaemic hospital admissions but none reported any increase related to the intervention. Insulin requirements were assessed in six trials [17, 18, 30, 32, 36, 38] but data were not suitable for a meta-analysis. The majority of trials reported no change in insulin regimen [17, 18, 38] or in the proportion of children who moved to pump therapy during the intervention [36]. Only two trials targeting groups of families reported a significant increase in insulin dose [32] or in frequency of insulin adjustment [30] in the intervention group. One trial assessed whether intervention increased children dietary adherence [33] but found no change. Finally, four trials assessed the impact of interventions on health service utilisation, including clinic visits [32, 35, 37], hospital admissions or contacts [18, 32, 37], and emergency hotline utilisation [35], but none found any significant change.

### Publication bias

Visual assessment of the funnel plot for HbA_1c_ showed a slightly asymmetric scatter which was mainly attributable to the presence of one small outlier study with positive effect (see S8 File).

## Discussion

We identified ten UK-based RCTs comparing psycho-educational interventions for improving management of T1D for CYP with a control group of usual care or attention control. Pooled data from nine of these trials showed that psycho-educational interventions conferred no glycaemic benefits over that achieved with standard care across the populations studied. The interventions used a wide variety of approaches, from purely educational programs to interventions combining educational with psychological components. Interventions with psychological components aiming to increase children’s self-efficacy to deal with diabetes appeared to show a moderate beneficial effect. However, evidence for an improvement in other important indicators of psychosocial functioning, such as quality of life, psychological distress and family functioning was absent.

In contrast to our findings on the synthesis of UK-based interventions, two recent meta-analyses mostly based on trials from North America [15, 16] reported significant glycaemic benefits of psycho-educational interventions in children and adolescents corresponding to reductions in HbA_1c_ by around half percentage point. They also provided evidence for significant psychological [15] and educational benefits [16]. There are a number of potential explanations for the discrepancies between our findings and that of previous reviews.

Firstly, previous reviews were mostly based on “efficacy” trials conducted in non-clinical settings by specialist interventionists with a solid background in psychology or psychiatry. In contrast, most of the interventions conducted in the UK were more pragmatic trials and delivered by non-specialist practitioners, mostly nurses and dietitians, after receiving relevant training. In fact, we found that only one UK intervention was delivered by a psychologist [31]; this was a person-centred intervention of motivational interviewing and showed the greatest beneficial effect in psychological outcomes, whilst also showing a trend for HbA_1c_ improvement. Two other UK interventions [32, 37] attempted to incorporate components of motivational interviewing into routine clinical practice by training non-psychologists, but showed no improvement on diabetes outcomes.

Some of the most successful psychological interventions in children with T1D have been delivered by persons with a background in psychology [42–46] which seems to suggest that the discipline, training and skills of the person delivering the intervention in a paediatric population could have an impact on outcomes. Evidence from interventions on adults with Type 2 Diabetes indicates that psychological and general health professionals are equally effective in delivering psychological interventions [47], but there is little evidence available for childhood T1D. Given the shortage of psychologists in the UK diabetes services [48], “efficacy” interventions may not be easily applied into routine clinical settings, yet it might be worthwhile to ensure that future interventions are delivered by rigorously trained personnel who have a sound understanding of both diabetes and psychological matters related to child teaching and learning.

Previous reviews also used different criteria for study selection, including trials in which the control group received care other than standard, including for example intensive insulin treatment or less intensive psychological treatment. One of the UK trials included in the current review [35] also included a third arm receiving both the psycho-educational intervention and intensive insulin therapy and found a significant reduction in HbA_1c_ by 1% as compared to standard care alone. Although a different design would be needed to disentangle the effect of the intervention from that of intensive therapy, this finding indicates that psycho-educational interventions could facilitate the uptake of intensive therapy schemes potentially enhancing their glycaemic benefits. Similar conclusions have been supported by USA trials [45, 49] which showed that psychological interventions used as an adjunct to intensive treatment conferred significant, consistent benefits in both glycaemic and psychosocial outcomes as compared to intensive treatment alone.

Although a lack of evidence for any glycaemic or psychosocial benefit of psycho-educational interventions conducted in the UK might simply reflect an absence of any “real” effect, there are other potential explanations for the negative findings. In most trials participation rates were poor which indicates that children entering trials might represent a population of children who already had a certain level of education and motivation in such a way that any additional intervention may not have a noticeable impact on their physical and psychological health (“ceiling effect”). Even the observed improvements in self-efficacy did not translate into glycaemic benefits, in most cases, measured one year after the end of the intervention. A longer duration of the intervention with provision of extended, continuous support even after the end of the program together with a longer follow-up period might be required for the behavioural changes to have an effect on the metabolic sequelae and translate into reductions in levels of HbA_1c_.

Findings of our review showed that most of the UK interventions are being offered to adolescents with more than one year duration of diabetes. This might be a potential reason for adolescents’ hesitance to participate as they tend to view such interventions as “non-essential”. Those individuals might have already established management strategies and behaviours that are difficult to challenge and change. Although targeting children with a shorter duration of diabetes can be challenging given the complex adaptation processes taking place, evidence from US trials suggests that implementation of psycho-educational programs earlier in the course of the disease can provide a more effective framework for such interventions [50, 51].

Low study enrolment and high withdrawal rates had also resulted in typically small sample sizes with only half of the UK trials having adequate power to detect an intervention effect. Since power calculations were mostly based on HbA_1c_, low sample size was particularly problematic for assessment of psycho-educational outcomes. Moreover, attendance rates were unsatisfactory and in some trials attendance was not considered sufficient to demonstrate any potential effect. Lack of intervention “reach” is a potentially important factor in the effectiveness of such interventions, and this may highlight the need to develop new and innovative strategies to decrease patient burden and encourage patient commitment in future interventions.

Educational and psychological interventions conducted in the UK also showed considerable heterogeneity in their content, intensity, selection of outcomes and delivery. This review highlights that although all of the interventions were theoretically grounded, they are poorly described, particularly with regard to the components of the intervention and the type of standard care, making it difficult to be replicated in practice. Attrition and reporting bias, especially with reference to psychosocial outcomes, was an issue in some studies and may further complicate interpretation of findings.

This is the first focused review to systematically examine the effectiveness of UK-based psycho-educational interventions on CYP with T1D. We used a rigorous protocol with high sensitivity and specificity to detect included studies. Psychosocial outcomes were grouped into conceptually homogeneous constructs, which allowed the examination of intervention effects across different aspects of psychosocial functioning. However, there are limitations. Firstly, our review was restricted to UK trials thus precluding us from making any direct comparisons between UK and non-UK interventions. Second, the variability in the scales used to measure psychological outcomes and the differences in follow-up between interventions have contributed to the observed heterogeneity across studies and warrant caution when interpreting the findings. Moreover, half of the included trials provided a single follow-up measurement which prevented us from meaningfully stratifying analyses by follow-up interval. We were also unable to assess the effect of interventions on long-term metabolic control since none of the included studies followed participants beyond two years. Third, the small number of studies did not allow us to formally examine potential modifiers, such as age, duration of diabetes and type of intervention. Fourth, the current review was limited to published studies. Although a comprehensive literature search was conducted and a number of “snowballing” techniques were used to identify eligible randomised trials, the potential of publication bias cannot be excluded. Finally, as per the eligibility criteria, we excluded one pilot trial of a UK intervention. Although some readers might consider this as a limitation, results of this small pilot study were in line with that of the subsequent main trial of the same intervention, which was incorporated into the current meta-analysis. Therefore, we believe that exclusion of this pilot study is unlikely to have affected our pooled estimates.

### Conclusion

There is currently insufficient evidence to recommend the use of psycho-educational programmes for children and adolescents with T1D in the UK. Successful implementation of similar interventions in the USA and other countries seems to suggest that such interventions are not inherently ineffective, and evaluation of their impact on diabetes outcomes requires focusing attention on the context within which these are applied and on potential target populations. One difference between UK trials and other non-UK successful trials has been the involvement of psychologists in the delivery of psychological interventions, which may be relevant to the deferring success observed. Future randomised controlled trials in the UK could potentially benefit by considering active involvement of psychological specialists in the delivery of psychologically informed interventions and provision of rigorous training of interventionists in psychological and clinical aspects of diabetes. Greater consideration could also be given to the early implementation of psycho-educational programs in newly diagnosed children and also to the provision of innovative strategies aiming to encourage patient engagement.

## Supporting information

S1 Checklist PRISMAchecklist. (DOC)

S1 File Prospero protocol. (PDF)

S2 File Search terms by database. (DOCX)

S3 File Data extraction form. (DOCX)

S4 File Sensitivity analysis. (DOCX)

S5 File Critical appraisal of RCTs included in the systematic review. (DOCX)

S6 File Outcomes of risk of bias assessment by trial. (DOCX)

S7 File Scales used to measure psycho-educational outcomes in included trials. (DOCX)

S8 File Funnel plot of intervention effects in HbA1c in the included studies. (DOCX)
