# Supplementary material for: Psycho-educational interventions for children and young people with Type 1 Diabetes in the UK: How effective are they? A systematic review and meta-analysis
Source: PLoS One. 2017 Jun 30;12(6):e0179685. doi: 10.1371/journal.pone.0179685 (PMC5493302; doi:10.1371/journal.pone.0179685)
Supplement: S1 File — (PDF) [file pone.0179685.s002.pdf]

Effectiveness of psycho-educational interventions for children and young people with type 1 diabetes in the UK

Dimitrios Charalampopoulos, Rakesh Amin, Russell Viner, Kathryn Hesketh, Terence Stephenson, Veena Mazarello Paes

Citation

Dimitrios Charalampopoulos, Rakesh Amin, Russell Viner, Kathryn Hesketh, Terence Stephenson, Veena Mazarello Paes. Effectiveness of psycho-educational interventions for children and young people with type 1 diabetes in the UK. PROSPERO 2015:CRD42015010701 Available from
http://www.crd.york.ac.uk/PROSPERO_REBRANDING/display_record.asp?ID=CRD42015010701

Review question(s)

What is the effectiveness of UK-based psycho-educational interventions in improving glycaemic control, diabetes management, knowledge and psychosocial functioning in children and young people (CYP) with type 1 diabetes (T1D)?

Are there any features in the delivery or content of the psycho-educational interventions conducted in the UK that are shown to be more effective than others?

Are there any harms or adverse effects (e.g. risk of hypoglycaemia or diabetes ketoacidosis) of psycho-educational interventions in CYP?

Searches

We searched the following databases: MEDLINE, EMBASE, Cochrane, PsycINFO, CINAHL, and Web of Science. We used a combination of free-text words and medical subject heading (MeSH) terms to generate 5 subsets of citations:

1. relating to population,

2. indexing the intervention,

3. for addressing the outcomes of interest,

4. for randomised controlled trials, and

5. for capturing studies conducted in the UK.

Results were then combined with “AND” and limited to children and young people up to 24 years and humans. The search was not limited by language or year of publication. In addition to searching electronic databases, a number of “snowballing” techniques were used to minimise the potential of publication bias and increase the sensitivity of our search (e.g. hand-searching reference lists of all selected articles, contacting experts and corresponding authors of selected articles).

Link to search strategy

http://www.crd.york.ac.uk/PROSPEROFILES/10701_STRATEGY_20150509.pdf

Types of study to be included

We only included randomised parallel controlled trials

Condition or domain being studied

Type 1 Diabetes (T1D) occurs as a result of an autoimmune destruction of the insulin-producing beta cells in the pancreas.This results in lack of insulin which in turn leads to increased blood glucose levels. T1D is one of the most

common chronic diseases in childhood and adolescence with about 28.2 new cases per 100,000 children under the age of 14 in the United Kingdom (UK).

The excess mortality among patients with T1D is mainly due to the long-term macro- and microvascular complications. Although these are of less immediate relevance to children as they occur later in adulthood, there is convincing evidence that glycaemic control, as measured by levels of glycated haemoglobin (HbA1c), is the most important modifiable risk factors for the development of diabetes complications.

The main goal of diabetes management is to optimise glycaemic control while maintaining quality of life. Although the mainstay of T1D management is through insulin and dietary modifications, the role of psychological and educational influences is well recognised. Commitment to a strict glycaemic control imposes significant psychological and emotional burdens on children and their families.

Participants/ population

We included RCT conducted in children and young people (up to and including the age of 24 years) with type 1 diabetes. Studies combining type 1 and type 2 diabetes or children (<25) with adults (>=25 years) were excluded unless the study design was stratified by type of diabetes or age group respectively.

Intervention(s), exposure(s)

We included trials conducted in the UK which examined the effectiveness of any type of educational or psycho-educational intervention in children and young people with type 1 diabetes. A broad definition of psycho-educational interventions was used including interventions targeting children and young people, families or health care professionals which aimed to improve management of diabetes in children. Eligible interventions could deal with any type of teaching diabetes-related knowledge or skills or/and provide any form of psychosocial training or support. Studies were not excluded based on setting, delivery or duration of the intervention, or outcome measured.

Comparator(s)/ control

We only included randomised parallel controlled trials (cluster or individual) which involved a non-intervention arm of children with T1D receiving “standard care”. Trials in which the control group was matched for the extra contact time (i.e. received additional support visits on top of standard care) were not excluded.

Outcome(s)

Primary outcomes

The primary outcome is glycaemic control, as measured by levels of glycated haemoglobin (HbA1c).

Secondary outcomes

Secondary outcomes included diabetes knowledge, psychosocial outcomes (including self-efficacy, quality of life, diabetes-related stress, and family functioning), service utilisation, self-management behaviours (i.e. frequency of glucose testing, and dietary choices), and adverse events (episodes of hypoglycaemia and diabetes ketoacidosis).

Data extraction, (selection and coding)

Titles and/or abstracts of studies retrieved using the search strategy were screened independently by two reviewers to identify studies that potentially meet the inclusion criteria mentioned above. The full text of these potentially eligible studies were retrieved and independently assessed for eligibility by two reviewers. Any disagreement between them were resolved through open discussion.

Extraction of data was developed based on guidelines by the Centre for Review and Dissemination (CRD) for systematic reviews in healthcare. We extracted data on study design and methodology, intervention characteristics including type, setting, provider, content, mode of delivery, duration and frequency of sessions, fidelity, attendance rates, co-interventions, and type of care received by controls. We also extracted data on number of participants randomised, recruitment and study completion rates, reasons for attrition, power of the study, participant demographics and characteristics at baseline. We finally extracted information on baseline and follow-up outcome data for each trial arm, and information for assessment of the risk of bias. For trials with multiple intervention arms, we extracted data for the intervention arm which was directly comparable to the control arm (i.e. without any co-intervention or change in routine care). In cross-over trial designs we only extracted data from the first period. Two

review authors extracted data independently, discrepancies were resolved through discussion.

Risk of bias (quality) assessment
Quality of individual trials will be assessed using six domains of the Cochrane Collaboration's tool for assessing risk of bias , including sequence generation, allocation concealment , blinding of outcome assessors, completeness of outcome data, selective reporting of outcomes, and other sources of bias. Since blinding of participants and personnel to knowledge of the intervention will not be possible, this domain will be excluded from the assessment. Any disagreement in risk classification between the two reviewers will be resolved by consensus.

Strategy for data synthesis
We will provide a narrative synthesis of the findings from the included studies, structured around the type of intervention, target population characteristics, type of outcome and intervention content. For continuous outcome measures we will use the standardised mean difference (SMD) to summarise intervention effects, calculated by dividing the between group difference in mean change-from-baseline scores (or follow-up scores adjusted for baseline values) by the pooled standard deviation of the change scores. We will calculated the intervention effect using the follow-up interval set a priori for the definition of the primary outcome or, if not stated, the most distal measurement with available data.For cluster-randomised trials we will use effect sizes adjusted for clustering effect and baseline values, or if not available, we will adjust sample sizes for the "design effect". Heterogeneity between the studies in effect measures will be assessed using the I-squared statistic. We will consider values of I-squared <50%, 50-75%, and >75% as indicative of low, moderate and high heterogeneity respectively. We will also assess evidence of publication bias. All analyses will be performed using STATA 12.

Analysis of subgroups or subsets
Where possible, we will investigate potential sources of heterogeneity by conducting subgroup analyses against a range of potential modifying factors, including study quality, type of intervention, and age group.

Dissemination plans
A manuscript will be submitted to a leading journal in this field.

Contact details for further information
Dr Charalampopoulos

UCL Institute of Child Health

30 Guilford Street

London WC1N 1EH

d.charalampopoulos@ucl.ac.uk

Organisational affiliation of the review
University College London, Institute of Child Health

http://www.ucl.ac.uk/ich

Review team
Dr Dimitrios Charalampopoulos, UCL Institute of Child Health
Dr Rakesh Amin, UCL Institute of Child Health
Professor Russell Viner, UCL Institute of Child Health
Dr Kathryn Hesketh, UCL Institute of Child Health
Professor Terence Stephenson, UCL Institute of Child Health
Mrs Veena Mazarello Paes, UCL Institute of Child Health

Collaborators
Dr David Taylor-Robinson, Department of Public Health and Policy, University of Liverpool, Liverpool, UK

Anticipated or actual start date
15 July 2014

Anticipated completion date
30 September 2015

Funding sources/sponsors
This is an independent report commissioned and funded by the Policy Research Programme in the Department of Health. The views expressed are not necessarily those of the Department.

Conflicts of interest
None known

Language
English

Country
England

Subject index terms status
Subject indexing assigned by CRD

Subject index terms
Child; Diabetes Mellitus, Type 1; Humans; Patient Education as Topic

Stage of review
Ongoing

Date of registration in PROSPERO
10 June 2015

Date of publication of this revision
10 June 2015

DOI
10.15124/CRD42015010701

Stage of review at time of this submission	Started	Completed
Preliminary searches	Yes	Yes
Piloting of the study selection process	Yes	Yes
Formal screening of search results against eligibility criteria	Yes	Yes
Data extraction	Yes	No
Risk of bias (quality) assessment	No	No
Data analysis	No	No

PROSPERO
International prospective register of systematic reviews
The information in this record has been provided by the named contact for this review. CRD has accepted this information in good faith and registered the review in PROSPERO. CRD bears no responsibility or liability for the content of this registration record, any associated files or external websites.
