# Supplementary material for: Psycho-educational interventions for children and young people with Type 1 Diabetes in the UK: How effective are they? A systematic review and meta-analysis
Source: PLoS One. 2017 Jun 30;12(6):e0179685. doi: 10.1371/journal.pone.0179685 (PMC5493302; doi:10.1371/journal.pone.0179685)
Supplement: S2 File — (DOCX) [file pone.0179685.s003.docx]

**Supplemental file S1: Search terms by database**

**Medline (Ovid)- 1946 to March Week 1 2016**

1. (("type 1" or "type I" or paediatric or pediatric or child* or young or youth* or juvenil* or (insulin adj depend*) or insulin-depend* or adolesc* or teen*) adj4 (diabet* or DM)).mp.
2. (T1DM or DM1 or T1D or IDDM).mp.
3. exp Diabetes Mellitus, Type 1/
4. or/1-3
5. (educat* or information or learn* or teach* or self-care or psycho* or counsel* or motivation* or famil* or parent*).mp.
6. ((“problem-solving” or cognitive or behavio* or CBT) adj4 (therap* or interv* or program* or train*)).mp.
7. Health Education/ or Psychotherapy/ or Cognitive Therapy/ or Behavior Therapy/
8. or/5-7
9. ((glycaemic or glycemic or metabolic or diabet* or glucose) adj4 (control or management or outcome* or level*)).mp.
10. (HbA1c or A1c or "HbA(1c)").mp.
11. ((insulin adj4 (use or injection* or dose*)).mp.
12. (Hypoglyc* or ketoacidosis or ketosis or DKA).mp.
13. (adher* or knowledge* or skill* or "insulin sensitivity" or behavi* or "quality of life" or manage* or self-management or control* or self-efficacy or diet* or eating or nutrition* or exerci* or regime).mp.
14. Hemoglobin A, Glycosylated/
15. Insulin resistance/
16. Quality of Life/
17. Health Behavior/
18. or/9-17
19. Randomized Controlled Trials as Topic/ or randomized controlled trial/ or Random Allocation/ or Double Blind Method/ or Single Blind Method/ or clinical trial/ or exp Clinical Trials as topic/or PLACEBOS/
20. clinical trial, phase i.pt or clinical trial, phase ii.pt or clinical trial, phase iii.pt or clinical trial, phase iv.pt or controlled clinical trial.pt or randomized controlled trial.pt or multicenter study.pt or clinical trial.pt
21. or/19-20
22. (clinical adj trial$).tw
23. ((singl$ or doubl$ or treb$ or tripl$) adj (blind$3 or mask$3)).tw
24. placebo$.tw or randomly allocated.tw
25. (allocated adj2 random$).tw
26. or/22-25
27. 21 or 26
28. case report.tw
29. letter/ or historical article/
30. or/28-29
31. 27 not 30
32. exp Great Britain/ or (Britain or british or Ireland or Irish or wales or welsh or Scottish or scots or Scotland or England or English or Birmingham or leeds or London or Liverpool or Manchester or Glasgow or Edinburgh or Cardiff or Belfast or Oxford or Cambridge or "United Kingdom" or UK or GB or aberdeen).ti,ab,in,cp,hw.
33. 4 AND 8 AND 18 AND 31 AND 32
34. limit 33 to (("all child (0 to 18 years)" or "young adult (19 to 24 years)") and humans)

**Embase(Ovid)- 1980 to 2016, week 11**

1. (("type 1" or "type I" or paediatric or pediatric or child* or young or youth* or juvenil* or (insulin adj depend*) or insulin-depend* or teen* or adolescen*) adj4 (diabet* or DM)).mp.
2. (T1DM or DM1 or T1D or IDDM).mp.
3. exp Diabetes Mellitus, Type 1/
4. or/1-3
5. (educat* or information or learn* or teach* or self-care or psycho* or counsel* or motivation*).mp.
6. (consult* adj4 skill* or famil* or parent*).mp.
7. ((“problem-solving” or cognitive or behavio* or CBT) adj4 (therap* or interv* or program* or train*)).mp.
8. Health Education/ or Psychotherapy/ or Cognitive Therapy/ or Behavior Therapy/
9. or/5-8
10. ((glycaemic or glycemic or metabolic or diabet* or glucose) adj4 (control or management or outcome* or level*)).mp.
11. (HbA1c or A1c or "HbA(1c)").mp.
12. (insulin adj4 (“use” or injection* or dose*)).mp.
13. (Hypoglyc* or ketoacidosis or ketosis or DKA).mp.
14. (adher* or knowledge* or skill* or behavi* or "quality of life" or manage* or self-management or control* or self-efficacy or diet* or eating or nutrition* or exerci* or regime).mp.
15. Hemoglobin A, Glycosylated/
16. Insulin resistance/ or Quality of Life/ or Health Behavior/
17. or/10-16
18. Clinical trial/ or Randomized controlled trial/ or Randomization/ or Single blind procedure/ or Double blind procedure/ or Crossover procedure/ or Placebo/ or Prospective study/
19. (Randomi?ed controlled trial$ or Rct or Random allocation or Randomly allocated or Allocated randomly or Single blind$ or Double blind$ or Placebo$).tw.
20. (allocated adj2 random).tw.
21. ((treble or triple) adj blind$).tw.
22. or/18-21
23. Case study/ or Abstract report/ or letter/ or Case report.tw.
24. 22 not 23
25. exp United Kingdom/ or (Britain or british or Ireland or Irish or wales or welsh or Scottish or scots or Scotland or England or English or Birmingham or leeds or London or Liverpool or Manchester or Glasgow or Edinburgh or Cardiff or Belfast or Oxford or Cambridge or "United Kingdom" or UK or GB or aberdeen).ti,ab,in,ad,cp,hw.
26. 4 and 9 and 17 and 24 and 25
27. limit 26 to (human and (child <unspecified age> or preschool child <1 to 6 years> or school child <7 to 12 years> or adolescent <13 to 17 years>))

**PsychINFO( Ovid) - 1806 to March Week 2 2016**

1. (("type 1" or "type I" or paediatric or pediatric or child* or young or youth* or juvenil* or (insulin adj depend*) or insulin-depend* or teen* or adolescen*) adj4 (diabet* or DM)).mp.
2. (educat* or information or learn* or teach* or self-care or psycho* or counsel* or motivation* or famil* or parent*).mp.
3. (consult* adj4 skill*).mp.
4. ((“problem-solving” or cognitive or behavio* or CBT) adj5 (therap* or interv* or program* or train*)).mp.
5. Health Education/ or Psychotherapy/ or Cognitive Therapy/ or Behavior Therapy/
6. or/2-5
7. ((glycaemic or glycemic or metabolic or diabet* or glucose) adj4 (control or management or outcome* or level*)).mp.
8. (HbA1c or A1c or "HbA(1c)").mp.
9. (insulin adj4 (“use” or injection* or dose*)).mp.
10. ((Hypoglyc* or ketoacidosis or ketosis or DKA) adj5 (admission* or episode*)).mp.
11. (adher* or knowledge* or skill* or behavi* or "quality of life" or manage* or self-management or control* or self-efficacy or diet* or eating or nutrition* or exerci* or regime).mp.
12. Hemoglobin A, Glycosylated/
13. Insulin resistance/ or Quality of Life/ or Health Behavior/
14. or/7-13
15. clinical trials/ or "treatment outcome clinical trial".md. or ((randomi?ed adj7 trial*) or ((single or doubl* or tripl* or treb*) and (blind* or mask*)) or (controlled adj3 trial*) or (clinical adj2 trial*)).ti,ab,id.
16. (Britain or british or Ireland or Irish or wales or welsh or Scottish or scots or Scotland or England or English or Birmingham or leeds or London or Liverpool or Manchester or Glasgow or Edinburgh or Cardiff or Belfast or Oxford or Cambridge or "United Kingdom" or UK or GB or aberdeen).ti,ab,in,hw,cq,lo.
17. 1 and 6 and 14 and 15 and 16
18. limit 17 to (human and (child <unspecified age> or preschool child <1 to 6 years> or school child <7 to 12 years> or adolescent <13 to 17 years>))

**CINAHL plus (EBSCOhost) - March 2016**

1. (MH "Diabetes Mellitus, Type 1+") OR (MH "Diabetes Mellitus+")
2. TX (("type 1" or paediatric or pediatric or child* or young or youth* or juvenile or adolescen*) n4 (diabet*))
3. TX (T1DM or DM1 or T1D)
4. 1 OR 2 OR 3
5. TX (educat* or information or learn* or teach* or self-care or psycho* or counsel* or motivation* or famil* or parent*)
6. TX (consult* n4 skill*)
7. TX ((“problem-solving” or cognitive or behavio* or CBT) n5 (therap* or interv* or program* or train*))
8. (MH "Health Education")
9. (MH "Psychotherapy") OR (MH "Cognitive Therapy")
10. (MH "Behavior Therapy") OR (MH "Behavior Modification")
11. 5 OR 6 OR 7 OR 8 OR 9 OR 10
12. TX ((glycaemic or glycemic or metabolic or diabet* or glucose) n4 (control or management or outcome* or level*))
13. TX (HbA1c or A1c or "HbA(1c)")
14. TX (insulin n4 (“use” or injection* or dose*))
15. TX ((Hypoglyc* or ketoacidosis or ketosis or DKA) n5 (admission* or episode*))
16. TX (adher* or knowledge* or skill* or behavi* or "quality of life" or manage* or self-management or control* or self-efficacy or diet* or eating or nutrition* or exerci* or regime)
17. (MH "Hemoglobin A, Glycosylated") OR (MH "Insulin Resistance") OR (MH "Glycemic Control")
18. (MH "Quality of Life") OR (MH "Health Behavior")
19. 12 OR 13 OR 14 OR 15 OR 16 OR 17 OR 18
20. (MH "Clinical Trials+")
21. PT Clinical trial
22. TX clinic* n1 trial*
23. TX ( (singl* n1 blind*) or (singl* n1 mask*) ) or TX ( (doubl* n1 blind*) or (doubl* n1 mask*) ) or TX ( (tripl* n1 blind*) or (tripl* n1 mask*) ) or TX ( (trebl* n1 blind*) or (trebl* n1 mask*) )
24. TX randomi* control* trial*
25. (MH "Random Assignment")
26. TX random* allocat*
27. TX placebo*
28. (MH "Placebos")
29. TX allocat* random*
30. 20 OR 21 OR 22 OR 23 OR 24 OR 25 OR 26 OR 27 OR 28 OR 29
31. 4 AND 11 AND 19 AND 30
32. limit 31 to (human and (child <unspecified age> or preschool child <1 to 6 years> or school child <7 to 12 years> or adolescent <13 to 17 years>))
33. limit 32 to uk & ireland

**Web of Science -databases: WOS OR CCC (Current Contents Connect) OR BCI (BIOSIS Citation Index) - March 2016**

1. TOPIC: ((("type 1" or "type I" or paediatric or pediatric or child* or young or youth* or juvenil* or (insulin NEAR depend*) or insulin-depend*) NEAR/4 (diabet* or DM))) OR TOPIC: ((T1DM or DM1 or T1D or IDDM))
2. TOPIC: ((educat* or information or learn* or teach* or self-care or psycho* or counsel* or motivation* or famil* or parent*)) OR TOPIC: ((consult* NEAR/4 skill*)) OR TOPIC: (((“problem-solving” or cognitive or behavio* or CBT) NEAR/4 (therap* or interv* or program* or train*)))
3. TOPIC: (((glycaemic or glycemic or metabolic or diabet* or glucose) NEAR/4 (control or management or outcome* or level*))) OR TOPIC: ((HbA1c or A1c or "HbA(1c)")) OR TOPIC: ((insulin NEAR/4 (“use” or injection* or dose*))) OR TOPIC: ((Hypoglyc* or ketoacidosis or ketosis or DKA)) OR TOPIC: ((adher* or knowledge* or skill* or behavi* or "quality of life" or manage* or self-management or control* or self-efficacy or diet* or eating or nutrition* or exerci* or regime))
4. TOPIC: (clinical trial* OR RCT* OR research design OR comparative stud* OR evaluation stud* OR controlled trial* OR follow-up stud* OR prospective stud* OR random* OR placebo* OR (single blind*) OR (double blind*))
5. 1 AND 2 AND 3 AND 4

Refined by: Databases: ( WOS OR CCC OR BCI ) AND COUNTRIES/TERRITORIES: ( NORTH IRELAND OR ENGLAND OR UK OR SCOTLAND OR WALES )

Timespan: All years.

Search language=Auto

**Cochrane - March 2016**

1. [mh "Diabetes Mellitus, Type 1"]

2. ("type 1" or "type I" or paediatric or pediatric or child* or young or youth* or juvenile or adolescen* or teen*) near/4 (diabet* or dm)

3. educat* or information or learn* or teach* or self-care or psycho* or counsel* or motivation*

4. [mh Psychotherapy] or [mh "health education"]

5. ("problem-solving" or cognitive or behavio* or CBT) near/5 (therap* or interv* or program* or train*) or (consult* near/4 skill*)

6. (glycaemic or glycemic or metabolic or diabet* or glucose) near/4 (control or management or outcome* or level*)

7. HbA1c or A1c or "HbA(1c)" or [mh hba1]

8. insulin near/4 ("use" or injection* or dose*)

9. (Hypoglyc* or ketoacidosis or ketosis or DKA) or [mh "insulin resistance"]

10. adher* or knowledge* or skill* or behavi* or "quality of life" or manage* or self-management or control* or self-efficacy or diet* or eating or nutrition* or exerci* or regime or [mh "health behavior"] or [mh "quality of life"]

11. #1 or #2

12. #3 or #4 or #5

13. #6 or #7 or #8 or #9 or #10

14. #11 and #12 and #13

15. adolescen* or youth* or young or child* or teen* or juvenil*

16. #14 and #15

17. [mh "united kingdom"] or [mh England] or [mh wales] or [mh scotland] or [mh "northern Ireland"] or (Britain or british or Ireland or Irish or wales or welsh or Scottish or scots or Scotland or England or English or Birmingham or leeds or London or Liverpool or Manchester or Glasgow or Edinburgh or Cardiff or Belfast or Oxford or Cambridge or "United Kingdom" or UK or GB or Aberdeen)

18. #16 and #17

Limit to trials
