# Supplementary material for: Psycho-educational interventions for children and young people with Type 1 Diabetes in the UK: How effective are they? A systematic review and meta-analysis
Source: PLoS One. 2017 Jun 30;12(6):e0179685. doi: 10.1371/journal.pone.0179685 (PMC5493302; doi:10.1371/journal.pone.0179685)
Supplement: S3 File — (DOCX) [file pone.0179685.s004.docx]

## Appendix B: Data Extraction Form

**DATA EXTRACTION FORM**

Date: ___________

Trial ID: ___________

**1. Bibliographical details**

First Author ________________ Publication year _________

**2. Details of study**

**General details**

Country _______________ recruitment period _____________________ Number of groups: _______

Inclusion criteria: __________________________________________________________________________

Exclusion criteria: __________________________________________________________________________

_________________________________________________________________________________________

**3. No of participants**

|  | **OVERALL** | Control group | Intervention group 1 | Intervention group 2 |
| --- | --- | --- | --- | --- |
| **Participation rate** (% of those contacted recruited): |  | | | |
| Numbers **randomized** |  |  |  |  |
| **Cross-overs** |  | C🡪I | I🡪C |  |
| Numbers **completed** study (time point= ) |  |  |  |  |
| Numbers **completed** study (time point= ) |  |  |  |  |
| Numbers **used in analyses** (time point= ) |  |  |  |  |
| Numbers **used in analyses** (time point= ) |  |  |  |  |
| **Post randomisation drop-outs** |  |  |  |  |
| **Post randomisation exclusions** |  |  |  |  |
| **Attrition rate** (% of those randomised completing study) |  |  |  |  |
| **Reasons for attrition explicitly reported?** 🗆 YES 🗆 NO, give details: | | | | |
| **Was sample size large enough to detect a meaningful difference if it had existed?** 🗆 YES 🗆 NO 🗆 can’t tell  Explain: | | | | |

**4. Participant characteristics at baseline**

| Age (mean, SD) |  |  |  |  |
| --- | --- | --- | --- | --- |
| Gender | ____M ____ F | ____M ____ F | ____M ____ F | ___M ___ F |
| Diabetes duration in years  (mean, SD) |  |  |  |  |
| Socio-economic status reported? Yes No give details: __________ |  |  |  |  |
| Ethnicity reported? Yes No |  |  |  |  |
| BMI (mean, SD) |  |  |  |  |
| INSULIN dose (u/kg/day) or other info |  |  |  |  |

- **Were the groups similar at baseline in all important characteristics**? 🗆 YES 🗆 NO 🗆 can’t tell
- **If groups not similar at baseline, was this adjusted for in the analyses**? 🗆 YES 🗆 NO 🗆 can’t tell

**5. Intervention**

**Type:** 🗆 **educational**

🗆 **Supportive Therapy** (incl. motivational interviewing, text messaging, solution focused therapy)

🗆 **CBT Therapy** (incl. goal setting, activity scheduling, problem solving, stress management)

🗆 **Family Therapy** 🗆 **Other** (eclectic approaches, training practitioners)

**Nature** (tick as many as apply):

🗆 Skills-training (e.g. insulin pump) 🗆 Diet-related 🗆 Exercise-related 🗆 Family-related 🗆 support

🗆 Stress reduction 🗆 problem-solving 🗆 goal-setting 🗆 shared agenda 🗆 solution-focused

🗆 Motivational/patient empowerment 🗆 other (specify) __________________________________________

**Explicit statement of theoretical base**? 🗆 NO 🗆 YES specify: _____________________________________

**Aim** of intervention: ________________________________________________________________________

**Overview** of intervention: ___________________________________________________________________ __________________________________________________________________________________________________________________________________________________________________________________

**Co-intervention**: ___________________________________________________________________________

| Was the intervention sufficiently **well described** **to be replicated** in practice? | Yes No Unclear |
| --- | --- |
| Was the intervention developed from a **pilot study**? | Yes No Unclear |
| Was intervention delivery **standardised**? | Yes No Unclear |
| Reference to where the **full trial protocol** can be accessed? | Yes No Unclear |

**Interventionist(s)**

🗆 Doctor 🗆 Psychologist 🗆 Nurse 🗆 Nutritionist/dietician 🗆 MDT member 🗆 not clear 🗆 other

Was the interventionist(s) **blinded**? 🗆 YES 🗆 NO 🗆 not stated

**Setting**

🗆 Diabetes clinic 🗆 Hospital (inpatient) 🗆 home 🗆 other community setting (__________) 🗆 Not stated

**Mode of delivery** (tick as many as apply)

🗆 Lecture 🗆 Interactive discussion/interview 🗆 Video 🗆 Printed information/leaflet 🗆 Computer

🗆 Not clear 🗆 other (specify) _____________________________

**Delivery of intervention**

🗆 Child 🗆 parent 🗆 Family or family group 🗆 clinician 🗆 other (__________________) 🗆 Not stated

**Time spent**

Duration of intervention _________ Time spent on each session _________ total No of sessions _________

**Fidelity**

Was the intervention sessions **delivered as planned** (e.g. consistent with protocol)? 🗆 YES 🗆 NO 🗆 can’t tell

Evidence for **training of interventionist(s)**? 🗆 YES 🗆 NO

How adherence to the protocol was **assessed/monitored**? _________________________________________

**Reach**

How many participants attended the sessions? __________________________________________________

Was it sufficient to demonstrate the effect of the intervention? 🗆 NO 🗆 YES 🗆 can’t tell

**6. Control & Maintenance**

Type of care received in controls: ____________________________________________________________

Advice for insulin/diet/exercise in both groups: _________________________________________________ **Aside from the intervention, were both groups treated equally?** 🗆 YES 🗆 NO 🗆 can’t tell

**7. Design**

🗆 single center 🗆 multi-center (specify) ________

***Method of randomisation***: ______________________________________________________________

***Unit of randomisation***: 🗆 individual 🗆 cluster (specify: _______________)

***Type of randomisation***: 🗆 simple 🗆 block (n= ____) 🗆 minimisation 🗆 stratified (by: ________________)

**Analysis**:

🗆 Intention-to-treat: *Patients are analysed with the group to which they were randomised* *regardless of the intervention they actually received. The outcome should be measured in all the patients (or imputed e.g. multiple imputation) and all the randomised participants should be included in the analysis.*

🗆 Available case analysis: *Patients are analysed with the group to which they were randomised (ITT principle)* ***but*** *only patients in whom the outcomes can be measured are included*

🗆 Per-protocol: *Patients who did* ***not receive*** *the intervention and who* ***crossed-over*** *to the other group are* ***excluded***

🗆 Treatment received analysis: *patients are analysed according to the treatment that they received irrespective of the group to which they were allocated*

🗆 Unclear

| Different endpoints/outcomes with different analysis? specify_______________________________________ |
| --- |

**8. Applicability**

| Have important populations been excluded from the study? *(consider disadvantaged populations, and possible differences in the intervention effect)* | Yes No Unclear |  |
| --- | --- | --- |

**9. Assessments** (specify whether from **start** *or* **end** of intervention)

🗆 Baseline 🗆 6 months 🗆 1 year 🗆 > 1 year (specify) ________________________________________

**Primary outcome**(s): ________________________________________________________________________

**Secondary outcomes**(s): _____________________________________________________________________

**Were any adverse outcomes measured?** 🗆 YES 🗆 NO, explain: ____________________________________

**Were appropriate statistical tests used to compare the groups?** (When applicable, was clustering adequately addressed in the analyses?) 🗆 YES 🗆 NO 🗆 can’t tell

**Outcome 1**

| **Outcome name** |  | |
| --- | --- | --- |
| **Time points measured** |  | |
| **Outcome definition** |  | |
| **Person measuring/reporting** |  | |
| **Unit of measurement**  *(if relevant)* |  | |
| **Scales: upper and lower limits** *(indicate whether high or low score is good)* |  | |
| **Is outcome/tool validated?** | Yes No Unclear |  |
| **Imputation of missing data** *(e.g. assumptions made for ITT analysis)* |  | |
| **Power** |  | |
| **Notes:** | | |

**Outcome 2**

| **Outcome name** |  | |
| --- | --- | --- |
| **Time points measured** |  | |
| **Outcome definition** |  | |
| **Person measuring/reporting** |  | |
| **Unit of measurement**  *(if relevant)* |  | |
| **Scales: upper and lower limits** *(indicate whether high or low score is good)* |  | |
| **Is outcome/tool validated?** | Yes No Unclear |  |
| **Imputation of missing data** *(e.g. assumptions made for ITT analysis)* |  | |
| **Power** |  | |
| **Notes:** | | |

**Outcome 3**

| **Outcome name** |  | |
| --- | --- | --- |
| **Time points measured** |  | |
| **Outcome definition** |  | |
| **Person measuring/reporting** |  | |
| **Unit of measurement**  *(if relevant)* |  | |
| **Scales: upper and lower limits** *(indicate whether high or low score is good)* |  | |
| **Is outcome/tool validated?** | Yes No Unclear |  |
| **Imputation of missing data** *(e.g. assumptions made for ITT analysis)* |  | |
| **Power** |  | |
| **Notes:** | | |

**Outcome 4**

| **Outcome name** |  | |
| --- | --- | --- |
| **Time points measured** |  | |
| **Outcome definition** |  | |
| **Person measuring/reporting** |  | |
| **Unit of measurement**  *(if relevant)* |  | |
| **Scales: upper and lower limits** *(indicate whether high or low score is good)* |  | |
| **Is outcome/tool validated?** | Yes No Unclear |  |
| **Imputation of missing data** *(e.g. assumptions made for ITT analysis)* |  | |
| **Power** |  | |
| **Notes:** | | |

**Outcome 5**

| **Outcome name** |  | |
| --- | --- | --- |
| **Time points measured** |  | |
| **Outcome definition** |  | |
| **Person measuring/reporting** |  | |
| **Unit of measurement**  *(if relevant)* |  | |
| **Scales: upper and lower limits** *(indicate whether high or low score is good)* |  | |
| **Is outcome/tool validated?** | Yes No Unclear |  |
| **Imputation of missing data** *(e.g. assumptions made for ITT analysis)* |  | |
| **Power** |  | |
| **Notes:** | | |

**Outcome 6**

| **Outcome name** |  | |
| --- | --- | --- |
| **Time points measured** |  | |
| **Outcome definition** |  | |
| **Person measuring/reporting** |  | |
| **Unit of measurement**  *(if relevant)* |  | |
| **Scales: upper and lower limits** *(indicate whether high or low score is good)* |  | |
| **Is outcome/tool validated?** | Yes No Unclear |  |
| **Imputation of missing data** *(e.g. assumptions made for ITT analysis)* |  | |
| **Power** |  | |
| **Notes:** | | |

**CONTINUOUS OUTCOMES**

| **OUTCOME 1** | **Time** | **Baseline**  **(mean and SD)** | **Post-intervention (mean and SD)** | **Change from baseline (mean and SD)** | **Other statistics** |
| --- | --- | --- | --- | --- | --- |
| IG-1 (n= ) |  |  |  |  |  |
| IG -2 (n= ) |  |  |  |  |  |
| CG (n= ) |  |  |  |  |  |
| **OUTCOME 2** | **Time** | **Baseline**  **(mean and SD)** | **Post-intervention (mean and SD)** | **Change from baseline (mean and SD)** | **Other statistics** |
| IG- 1 (n= ) |  |  |  |  |  |
| IG -2 (n= ) |  |  |  |  |  |
| CG (n= ) |  |  |  |  |  |
| **OUTCOME 3** | **Time** | **Baseline**  **(mean and SD)** | **Post-intervention (mean and SD)** | **Change from baseline (mean and SD)** | **Other statistics** |
| IG- 1 (n= ) |  |  |  |  |  |
| IG -2 (n= ) |  |  |  |  |  |
| CG (n= ) |  |  |  |  |  |
| **OUTCOME 4** | **Time** | **Baseline**  **(mean and SD)** | **Post-intervention (mean and SD)** | **Change from baseline (mean and SD)** | **Other statistics** |
| IG- 1 (n= ) |  |  |  |  |  |
| IG -2 (n= ) |  |  |  |  |  |
| CG (n= ) |  |  |  |  |  |
| **OUTCOME 5** | **Time** | **Baseline**  **(mean and SD)** | **Post-intervention (mean and SD)** | **Change from baseline (mean and SD)** | **Other statistics** |
| IG- 1 (n= ) |  |  |  |  |  |
| IG -2 (n= ) |  |  |  |  |  |
| CG (n= ) |  |  |  |  |  |

| **OUTCOME 6** | **Time** | **Baseline**  **(mean and SD)** | **Post-intervention (mean and SD)** | **Change from baseline (mean and SD)** | **Other statistics** |
| --- | --- | --- | --- | --- | --- |
| IG-1 (n= ) |  |  |  |  |  |
| IG -2 (n= ) |  |  |  |  |  |
| CG (n= ) |  |  |  |  |  |
| **OUTCOME 7** | **Time** | **Baseline**  **(mean and SD)** | **Post-intervention (mean and SD)** | **Change from baseline (mean and SD)** | **Other statistics** |
| IG- 1 (n= ) |  |  |  |  |  |
| IG -2 (n= ) |  |  |  |  |  |
| CG (n= ) |  |  |  |  |  |
| **OUTCOME 8** | **Time** | **Baseline**  **(mean and SD)** | **Post-intervention (mean and SD)** | **Change from baseline (mean and SD)** | **Other statistics** |
| IG- 1 (n= ) |  |  |  |  |  |
| IG -2 (n= ) |  |  |  |  |  |
| CG (n= ) |  |  |  |  |  |
| **OUTCOME 9** | **Time** | **Baseline**  **(mean and SD)** | **Post-intervention (mean and SD)** | **Change from baseline (mean and SD)** | **Other statistics** |
| IG- 1 (n= ) |  |  |  |  |  |
| IG -2 (n= ) |  |  |  |  |  |
| CG (n= ) |  |  |  |  |  |
| **OUTCOME 10** | **Time** | **Baseline**  **(mean and SD)** | **Post-intervention (mean and SD)** | **Change from baseline (mean and SD)** | **Other statistics** |
| IG- 1 (n= ) |  |  |  |  |  |
| IG -2 (n= ) |  |  |  |  |  |
| CG (n= ) |  |  |  |  |  |

**BINARY OUTCOMES**

| **OUTCOME 1** | **Time** | **Total number of participants** | **No of events** |
| --- | --- | --- | --- |
| IG-1 |  |  |  |
| IG -2 |  |  |  |
| CG |  |  |  |
| **OUTCOME 2** | **Time** | **Total number of participants** | **No of events** |
| IG-1 |  |  |  |
| IG -2 |  |  |  |
| CG |  |  |  |
| **OUTCOME 3** | **Time** | **Total number of participants** | **No of events** |
| IG-1 |  |  |  |
| IG -2 |  |  |  |
| CG |  |  |  |
| **OUTCOME 4** | **Time** | **Total number of participants** | **No of events** |
| IG-1 |  |  |  |
| IG -2 |  |  |  |
| CG |  |  |  |

**Cochrane Collaboration’s tool for assessing risk of bias**

| **Domain** | **Risk of bias** | | | **Support for judgement** | **Location in text**  *(pg & ¶/fig/table)* |
| --- | --- | --- | --- | --- | --- |
|  | Low risk | High risk | Unclear |  |  |
| **Random sequence generation**  *(selection bias)* |  |  |  |  |  |
| **Allocation concealment**  *(selection bias)* |  |  |  |  |  |
| **Blinding of outcome assessment**  *(detection bias)* |  |  |  | **Outcome group:**  🗆 This information was not available  🗆 Lack of blinding will not result in bias for this outcome |  |
| *(if required)* |  |  |  | **Outcome group:** |  |
| **Incomplete outcome data**  *(attrition bias)* |  |  |  | **Outcome group:**  🗆 There were no post-randomisation drop-outs  🗆 There were post-randomisation drop-outs  🗆 This information was not available |  |
| **Incomplete outcome data**  *(attrition bias)* |  |  |  | **Outcome group:**  🗆 There were no post-randomisation drop-outs  🗆 There were post-randomisation drop-outs  🗆 This information was not available |  |
| **Selective outcome reporting?**  *(reporting bias)* |  |  |  | 🗆 All important outcomes were reported.  🗆 Some important outcomes which will generally be assessed were not reported |  |
| **Other bias** |  |  |  |  |  |
| **Notes:** | | | | | |
