# Supplementary material for: Psycho-educational interventions for children and young people with Type 1 Diabetes in the UK: How effective are they? A systematic review and meta-analysis
Source: PLoS One. 2017 Jun 30;12(6):e0179685. doi: 10.1371/journal.pone.0179685 (PMC5493302; doi:10.1371/journal.pone.0179685)
Supplement: S4 File — (DOCX) [file pone.0179685.s005.docx]

**Supplemental file S3: Sensitivity analysis - Meta-analyses using the shortest available follow-up measurement**

**Random effects meta-analysis of change scores in HbA1c (%) in psycho-educational intervention group compared with control group. Intervention effects calculated as Standardised Mean Difference (SMD) with 95% confidence interval. A negative effect indicates improved glycaemic control** **attributable to intervention.**

**Intervention effects on psychosocial outcomes calculated as Standardised Mean Difference (SMD) of change scores with 95% confidence interval. A positive effect in quality of life, self-efficacy, and family functioning and a negative effect is psychological distress favour intervention. The diamonds show the pooled SMD based on random effects**
