# Supplementary material for: Psycho-educational interventions for children and young people with Type 1 Diabetes in the UK: How effective are they? A systematic review and meta-analysis
Source: PLoS One. 2017 Jun 30;12(6):e0179685. doi: 10.1371/journal.pone.0179685 (PMC5493302; doi:10.1371/journal.pone.0179685)
Supplement: S5 File — (DOCX) [file pone.0179685.s006.docx]

Supplemental file S2. Critical appraisal of RCTs included in the systematic review

| First author (publication year) | Participation rate^a^ | Type of analysis | Statistician blinded? | Retention rate ^b^ in each arm (CG/ IG) | Reasons for attrition explicitly reported | Groups similar at baseline? | Sample size large enough to detect a meaningful effect if it had existed? | Intervention sufficiently described to be replicated | Reference to full trial protocol | Have important populations been excluded? | Intervention delivered as planned? | Evidence for training of interventionist? | Was adherence to the protocol monitored? | Attendance | Was attendance sufficient to demonstrate effect ^c^ ? |  |
| --- | --- | --- | --- | --- | --- | --- | --- | --- | --- | --- | --- | --- | --- | --- | --- | --- |
| Bloomfield (1990) | 52% | ITT | NR | 100% / 100% | NA | Y | ? ^d^ | N | N | N | NR | N | NR | Attendance rate >80% | Y |  |
| Howells (2002) | 65% | ITT ^i^ | NR | 90.3% / 83.9% | Y | Y | Y ^e^ | N | N | N | Y | Y | Y | Each participant received an average number of 16 phone calls | Y |  |
| Franklin (2006) | 70% | ITT ^i^ | NR | 96.4% / 96.7% | Y | Y | N ^f^ | Y | Y | N | Y | N | NR | NA | Y |  |
| Channon (2007) | 47% | ITT ^i^ | NR | 54% / 69.8% | N | Y | N | N | N | Y ^g^ | ? | N | Y | NR | ? |  |
| Murphy (2012) | 37% | ITT ^i^ | NR | 95.9% / 97.5% | Y | Y | Y | Y | Y | N | ? | Y | NR | 50% of participants attended ≥ 4/6 sessions, 30% attended none | N |  |
| Robling (2012) | 55% | ITT ^i^ | NR | 95.2% / 95.3% | N | N | Y | N | Y | N | N | Y | Y | Intervention incorporated into routine clinical care | Y |  |
| Coates (2013) | 34% | ITT | NR | 43.1% / 44.3% | N | ? | N | N | Y | ? | ? | N | NR | 94% of participant completed training | Y |  |
| Doherty (2013) | NA ^j^ | ITT ^i^ | NR | 69.6% / 50% | Y | N | N | N | Y | ? | Y | N | Y | participants completed an average of 6.5/10 modules | N |  |
| Christie (2014) | 31% | ITT ^i^ | NR | 81.4% / 74.2% | Y | Y | Y | Y | Y | Y ^h^ | Y | Y | Y | 37% of families did not attend any module | N |  |
| Price (2016) | 27% | ITT ^i^ | NR | 82.4% / 72.5% | Y | Y | N | Y | Y | N | Y | Y | Y | 29 out of 995 course days (3%) missed | Y |  |
| *Notes: ITT: Intention-to-treat, Y: Yes, N: No, NR: Non-Reported, NA: not applicable,?: unclear*  *^a^ % of eligible participants contacted recruited*  *^b^ % of those randomised completing study (it refers to the primary outcome measured at the longest interval)*  *^c^ judgement reached by reviewers after consideration of attendance information and trial authors’ interpretation in the manuscript*  *^d^ no power calculations made*  *^e^ adequate power for psychological outcomes but not for HbA_1c_*  *^f^ an unreasonably high difference in HbA_1c_ was assumed for power calculations (1.7%)*  ^g^ *non-white children*  ^h^ *children with hba_1c_ < 8.5%*  *^i^ only patients in whom the outcomes were measured have been included in the analysis*  *^j^ web-based trial* | | | | | | | | | | | | | | | | |
