# Supplementary material for: Psycho-educational interventions for children and young people with Type 1 Diabetes in the UK: How effective are they? A systematic review and meta-analysis
Source: PLoS One. 2017 Jun 30;12(6):e0179685. doi: 10.1371/journal.pone.0179685 (PMC5493302; doi:10.1371/journal.pone.0179685)
Supplement: S6 File — (DOCX) [file pone.0179685.s007.docx]

Supplemental file S3. Outcomes of risk of bias assessment by trial

| First author (publication year) | Selection bias:  random sequence generation | Selection bias:  allocation concealment | Detection bias: blinding of outcome assessment | | Attrition bias: incomplete outcome data | | Reporting bias: selective reporting | Other bias |
| --- | --- | --- | --- | --- | --- | --- | --- | --- |
|  |  |  | Hba_1c_ | PEO | Hba_1c_ | PEO |  |  |
| Bloomfield (1990) | ? | ? | - | ? | - | - | + | + ^a^ |
| Howells (2002) | - | - | - | ? | - | - | - | - |
| Franklin (2006) | - | ? | - | - | - | - | - | - |
| Channon (2007) | ? | - | - | - | + | + | + | - |
| Murphy (2012) | - | ? | - | ? | - | + | - | - |
| Robling (2012) | ? | - | - | - | - | - | + | - |
| Coates (2013) | ? | - | - | ? | ? ^d^ | ? ^d^ | + | - |
| Doherty (2013) | - | - | NA | - | NA | + | - | + ^b^ |
| Christie (2014) | ? | ? | - | - | - | - ^c^ | - | - |
| Price (2016) | - | - | - | - | - | - | + | - |
| *Note: PEO: psycho-educational outcomes, + indicates high risk of bias, - indicates low risk of bias, and ? indicates unclear risk of bias*  *^a^ cross-over design inappropriate*  *^b^ baseline imbalance*  *^c^ high risk for knowledge only*  *^d^ insufficient information for reasons of drop-outs and methods of imputation for missing values* | | | | | | | | |
