# Supplementary material for: Psycho-educational interventions for children and young people with Type 1 Diabetes in the UK: How effective are they? A systematic review and meta-analysis
Source: PLoS One. 2017 Jun 30;12(6):e0179685. doi: 10.1371/journal.pone.0179685 (PMC5493302; doi:10.1371/journal.pone.0179685)
Supplement: S7 File — (DOCX) [file pone.0179685.s008.docx]

| **Supplemental file S4: Scales used to measure psycho-educational outcomes in included trials** | | |
| --- | --- | --- |
| **First author (publication year)** | **Educational outcomes** | **Psychosocial outcomes** |
| Bloomfield (1990) | Diabetes knowledge scale (1) | Strandford-Binet/Wechesler Intelligence scales^¥^ (no ref)  Rutter Behavior Scale (2)^¥^  Social Maturity Scale (3)^¥^  Child responsibility in diabetes management & family life^¥^ (no ref) |
| Howells (2002) | Diabetes knowledge scale (DKN) (4) | Self-efficacy for diabetes (SED) (5)  Environmental barriers to adherence questionnaire (BA) (6)  Social problem solving inventory (PS) (7) |
| Franklin (2006) | Diabetes knowledge scale (DKN) (4) | Self-efficacy for diabetes (SED) (5)  Diabetes Social Support interview (8) |
| Channon (2007) | Diabetes knowledge scale (DKN) (1) | Self-efficacy for diabetes (SED) (5)  Diabetes Quality of life measure for youths (DQoLY) (9)  Child health locus of control (CHLC) (10)  Health care climate questionnaire (HCCQ) (11)  Well-being questionnaire (WBQ) (12)  Personal models of diabetes scale (PMDQ) (13)  Diabetes Family Behavior scale (DFBS) (14) |
| Murphy (2012) |  | Diabetes Quality of life measure for youths- short form (DQoLY) (15)  WHO Health Behavior in School Children (HBSC) (16)  Problem Areas in Diabetes scale (PAID) (17)  Diabetes Family Responsibility Questionnaire (shorten form) (DFR) (18) |
| Robling (2012) |  | Paediatric quality of life inventory: diabetes module (PedsQoL) (19)  Patient enablement instrument (PEI) (20)  Health care climate questionnaire (HCCQ) (11)  Problem Areas in Diabetes scale (PAID) (17)  Diabetes continuity of care scale (21)  Clinic-related affect (not validated)  Importance & confidence in diabetes management (not validated) |
| Coates (2013) |  | Perception of control (no ref)  Patient empowerment (no ref)  Diabetes Quality of life measure for youths (DQoLY) (9)  Diabetes care profile (DCP) (no ref) |
| Doherty (2013) |  | Diabetes family conflict scale (DFCS) (22)  Paediatric Inventory for parents (PIP) (23)  Eyberg child behavior inventory (ECBI) (24)  Parenting scale (PS) (25)  Parenting sense of competence scale (PSOC) (26) |
| Christie (2014) | Diabetes Knowledge and skills (27) | Paediatric quality of life inventory: generic (PedsQoL-G) and diabetes module (PedsQoL-D) (19)  Strengths and difficulties Questionnaire- impact score (SDQ) (28)  Diabetes Family Responsibility Questionnaire (DFRQ) (29)  Self-efficacy for diabetes (SED) (5) |
| Price (2016) |  | Paediatric quality of life inventory: diabetes module (PedsQoL-D) (19)  Paediatric quality of life inventory: generic (PedsQoL- G) (30)  Self-efficacy for Diabetes (5) |
| ^¥^ Results not reported by authors | | |

**References**

1. Dunn SM, Bryson JM, Hoskins PL, Alford JB, Handelsman DJ, Turtle JR. Development of the diabetes knowledge (DKN) scales: forms DKNA, DKNB, and DKNC. Diabetes care. 1984;7(1):36-41.

2. Ruller M, Tizard, J., Whitmore, K. . Education, Health, and Behaviour. Longman, editor. London1970.

3. Doll EA, Vineland, Training S, American Guidance S. Vineland social maturity scale; condensed manual of directions. Circle Pines, Minn.: American Guidance Service; 1965.

4. Beeny L.J. DSM, Welch G. Measurement of diabetes knowledge-the development of the DKN scales. New York: Psychology Press; 1994. 159-91 p.

5. Grossman HY, Brink S, Hauser ST. Self-efficacy in adolescent girls and boys with insulin-dependent diabetes mellitus. Diabetes care. 1987;10(3):324-9.

6. Irvine AA, Saunders JT, Blank MB, Carter WR. Validation of scale measuring environmental barriers to diabetes-regimen adherence. Diabetes care. 1990;13(7):705-11.

7. D'Zurilla TJ, Goldfried MR. Problem solving and behavior modification. Journal of abnormal psychology. 1971;78(1):107-26.

8. La Greca AM, Auslander WF, Greco P, Spetter D, Fisher EB, Jr., Santiago JV. I get by with a little help from my family and friends: adolescents' support for diabetes care. Journal of pediatric psychology. 1995;20(4):449-76.

9. Ingersoll GM, Marrero DG. A modified quality-of-life measure for youths: psychometric properties. The Diabetes educator. 1991;17(2):114-8.

10. Wallston BS, Wallston KA, Kaplan GD, Maides SA. Development and validation of the health locus of control (HLC) scale. Journal of consulting and clinical psychology. 1976;44(4):580-5.

11. Williams GC, Freedman ZR, Deci EL. Supporting autonomy to motivate patients with diabetes for glucose control. Diabetes care. 1998;21(10):1644-51.

12. C B. The Well-Being Questionnaire. Switzerland: Harwood Academic Publishers; 1994.

13. Hampson SE, Glasgow RE, Foster LS. Personal models of diabetes among older adults: relationship to self-management and other variables. The Diabetes educator. 1995;21(4):300-7.

14. McKelvey J, Waller DA, North AJ, Marks JF, Schreiner B, Travis LB, et al. Reliability and validity of the Diabetes Family Behavior Scale (DFBS). The Diabetes educator. 1993;19(2):125-32.

15. Skinner TC, Hoey H, McGee HM, Skovlund SE, Hvidore Study Group on Childhood D. A short form of the Diabetes Quality of Life for Youth questionnaire: exploratory and confirmatory analysis in a sample of 2,077 young people with type 1 diabetes mellitus. Diabetologia. 2006;49(4):621-8.

16. Currie C. HK, Settertobulte W., Smith R., Todd J. health behaviour in School-aged Children. Geneve.

17. Polonsky WH, Anderson BJ, Lohrer PA, Welch G, Jacobson AM, Aponte JE, et al. Assessment of diabetes-related distress. Diabetes care. 1995;18(6):754-60.

18. Cameron FJ, Skinner TC, de Beaufort CE, Hoey H, Swift PG, Aanstoot H, et al. Are family factors universally related to metabolic outcomes in adolescents with Type 1 diabetes? Diabetic medicine : a journal of the British Diabetic Association. 2008;25(4):463-8.

19. Varni JW, Burwinkle TM, Jacobs JR, Gottschalk M, Kaufman F, Jones KL. The PedsQL in type 1 and type 2 diabetes: reliability and validity of the Pediatric Quality of Life Inventory Generic Core Scales and type 1 Diabetes Module. Diabetes care. 2003;26(3):631-7.

20. Howie JG, Heaney DJ, Maxwell M, Walker JJ. A comparison of a Patient Enablement Instrument (PEI) against two established satisfaction scales as an outcome measure of primary care consultations. Family practice. 1998;15(2):165-71.

21. Dolovich LR, Nair KM, Ciliska DK, Lee HN, Birch S, Gafni A, et al. The Diabetes Continuity of Care Scale: the development and initial evaluation of a questionnaire that measures continuity of care from the patient perspective. Health & social care in the community. 2004;12(6):475-87.

22. Hood KK, Butler DA, Anderson BJ, Laffel LM. Updated and revised Diabetes Family Conflict Scale. Diabetes care. 2007;30(7):1764-9.

23. Streisand R, Braniecki S, Tercyak KP, Kazak AE. Childhood illness-related parenting stress: the pediatric inventory for parents. Journal of pediatric psychology. 2001;26(3):155-62.

24. Eyberg S, Pincus, D. . ECBI & SESBI-R: Eyberg Child Behavior Inventory and Sutter-Eyberg Student Behavior Inventory-Revised : professional manual: Psychological Assessment Resources 1999.

25. Arnold DS, O'Leary SG, Wolff LS, Acker MM. The Parenting Scale: A measure of dysfunctional parenting in discipline situations. Psychological Assessment. 1993;.5(2):pp.

26. Johnston C, Mash EJ. A measure of parenting satisfaction and efficacy. Jun 1989. Journal of Clinical Child Psychology. 1989;.18(2):pp.

27. Kaufman FR, Austin J, Lloyd J, Halvorson M, Carpenter S, Pitukcheewanont P. Characteristics of glycemic control in young children with type 1 diabetes. Pediatric diabetes. 2002;3(4):179-83.

28. Goodman R. The Strengths and Difficulties Questionnaire: a research note. Journal of child psychology and psychiatry, and allied disciplines. 1997;38(5):581-6.

29. Anderson BJ, Auslander WF, Jung KC, Miller JP, Santiago JV. Assessing family sharing of diabetes responsibilities. Journal of pediatric psychology. 1990;15(4):477-92.

30. Varni JW, Seid M, Kurtin PS. PedsQL 4.0: reliability and validity of the Pediatric Quality of Life Inventory version 4.0 generic core scales in healthy and patient populations. Med Care. 2001;39(8):800-12.
