# Supplementary figures and images for: Psycho-educational interventions for children and young people with Type 1 Diabetes in the UK: How effective are they? A systematic review and meta-analysis
Source: PLoS One. 2017 Jun 30;12(6):e0179685. doi: 10.1371/journal.pone.0179685 (PMC5493302; doi:10.1371/journal.pone.0179685)

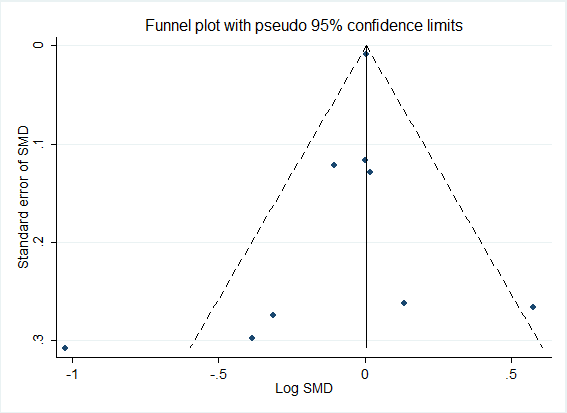


Funnel plot of intervention effects in HbA1c in the included studies

Supplement: S8 File — (DOCX) [file pone.0179685.s009.docx]
